# Pyramiding of scald resistance genes in four spring barley MAGIC populations

**DOI:** 10.1007/s00122-021-03930-y

**Published:** 2021-08-04

**Authors:** Juho Hautsalo, Fluturë Novakazi, Marja Jalli, Magnus Göransson, Outi Manninen, Mika Isolahti, Lars Reitan, Stein Bergersen, Lene Krusell, Charlotte Damsgård Robertsen, Jihad Orabi, Jens Due Jensen, Ahmed Jahoor, Therése Bengtsson, the PPP Barley Consortium, Merja Veteläinen, Outi Manninen, Mika Isolahti, Muath Alsheikh, Stein Bergersen, Constantin Jansen, Susanne Windju, Lars Reitan, Marja Jalli, Juho Hautsalo, Ahmed Jahoor, Jihad Orabi, Nana Vangdorf, Jens Due Jensen, Lene Krusell, Rasmus Lund Hjortshøj, Charlotte Damsgård Robertsen, Ahmed Jahoor, Therése Bengtsson, Fluturë Novakazi, Inger Åhman, Magnus Göransson, Hrannar Smári Hilmarsson, Sæmundur Sveinsson

**Affiliations:** 1 Natural Resources Institute Finland (Luke), Survontie 9, 40500 Jyväskylä, Finland; 2 Department of Plant Breeding, Swedish University of Agricultural Sciences, P.O. Box 190, 234 22 Lomma, Sweden; 3 Natural Resources Institute Finland (Luke), Tietotie 4, 31600 Jokioinen, Finland; 4 Faculty of Land and Animal Resources, The Agricultural University of Iceland, Hvanneyri, 311 Borgarnes Iceland; 5 Boreal Plant Breeding Ltd., Myllytie 10, 31600 Jokioinen, Norway; 6 Graminor Ltd. Hommelstadvegen 60, 2322 Ridabu, Norway; 7 Sejet Plant Breeding, Nørremarksvej 67, 8700 Horsens, Norway; 8 Nordic Seed A/S, Kornmarken 1, 8464 Galten, Denmark

**Keywords:** *Rhynchosporium commune*, GWAS, *Hordeum vulgare* L., Multi-parent Advanced Generation Inter-Cross, FarmCPU, BLINK

## Abstract

Genome-Wide Association Studies (GWAS) of four Multi-parent Advanced Generation Inter-Cross (MAGIC) populations identified nine regions on chromosomes 1H, 3H, 4H, 5H, 6H and 7H associated with resistance against barley scald disease. Three of these regions are putatively novel resistance Quantitative Trait Loci (QTL). Barley scald is caused by *Rhynchosporium commune*, one of the most important barley leaf diseases that are prevalent in most barley-growing regions. Up to 40% yield losses can occur in susceptible barley cultivars. Four MAGIC populations were generated in a Nordic Public–Private Pre-breeding of spring barley project (PPP Barley) to introduce resistance to several important diseases. Here, these MAGIC populations consisting of six to eight founders each were tested for scald resistance in field trials in Finland and Iceland. Eight different model covariate combinations were compared for GWAS studies, and the models that deviated the least from the expected p-values were selected. For all QTL, candidate genes were identified that are predicted to be involved in pathogen defence. The MAGIC progenies contained new haplotypes of significant SNP-markers with high resistance levels. The lines with successfully pyramided resistance against scald and mildew and the significant markers are now distributed among Nordic plant breeders and will benefit development of disease-resistant cultivars.

**Supplementary Information:**

The online version of this article (10.1007/s00122-021-03930-y) contains supplementary material, which is available to authorized users.

## Introduction

Barley (*Hordeum vulgare* L.) is the most widely grown cereal in the Nordic countries, and with 159 million tonnes produced from 51 million hectares globally, it is also the fourth most widely produced cereal in the world. Most of the barley harvest is used as feed and food, and approximately 20% of worldwide barley production is used by the malting industry (FAOSTAT 2020).

Fungal pathogens cause significant yield losses in barley production and *Rhynchosporium commune* Zaffarano, McDonald and Linde sp. nov. (formerly: *R. secalis* (Oudem.) J.J. Davis) (Zaffarano et al. 2011) is one of the most important diseases worldwide (Avrova and Knogge 2012; Zhang et al. 2020). The name *R. graminicola* Heinsen 1897 is also suggested to be used as a replacing synonym for *R. commune* due to its longer history (Crous et al. 2021). *R. commune* is the causal agent of barley scald, also known as leaf scald, leaf blotch or *Rhynchosporium*. In susceptible cultivars, the yield losses can be up to 40% and yield quality can be severely decreased (Paulitz and Steffenson 2011).

*R. commune* has several disease cycles during the growing season. The disease enters fields either through crop residue of previous crops or infected seeds (Davis and Fitt 1992), and further disease cycles are formed by conidia spreading by splash dispersal from infected leaves (Fitt et al. 1986). Inoculum load can be reduced by agronomic practices like tillage and crop rotation (Arvidsson 1998). Scald can also be controlled by using fungicides, but the pathogen is shown to develop resistant strains against frequently used active ingredients (Avrova and Knogge 2012). In addition to fungicides, cultivar resistance is an effective way of providing protection against initial infection and managing the disease in a sustainable manner (McLean and Hollaway 2018).

So far, 148 quantitative trait loci (QTL) for scald resistance have been reported in barley (reviewed in Zhang et al. 2020) including the following major resistance genes (R-genes): *Rrs14* on chromosome 1H, *Rrs17* on 2H, *Rrs1* and *Rrs4* on 3H, *Rrs16* on 4H, *Rrs13* and *Rrs18* on 6H, *Rrs2, Rrs 12*, *Rrs15*, on 7H. *R. commune* is a genetically highly diverse pathogen (Abang et al. 2006; McDonald 2015), which emphasizes the importance of using partial resistance over single R-genes and also the need to find new resistance mechanisms to replace the ones that are already overcome by pathogen evolution. For example, the effector protein NIP1, which is the product of the *R. commune* avirulence gene *AvrRrs1* (Rohe et al. 1995), is recognized by cultivars carrying the major R-gene *Rrs1.* This would induce the expression of pathogenesis‐related 10 gene in leaves (Steiner‐Lange et al. 2003), but this defence reaction can be prevented by mutations occurring in NIP1 (van’t Slot et al. 2007). R-genes trigger plant defence responses by directly or indirectly recognizing the products of avirulence genes expressed by the pathogen during infection. However, due to the simple genetic architecture of this interaction, major gene-mediated resistance can be broken down after only a short period of commercial cultivation (Abang et al. 2006), unless the avirulence gene product is essential to the pathogen. Partial resistance is shown to reduce scald severity (Williams and Owen 1975; Kari and Griffiths 1993; Looseley et al. 2012). Quantitative enhancement of partial resistance is possible (Niks et al. 2011), and incorporation of major R-genes can also be applied in the process to reach a level of resistance where severe yield losses and deterioration of yield quality can be avoided. However, pyramiding several genes in breeding lines that may have similar or additive phenotypic responses depending on the environment and pathogen isolates is difficult. Thus, the development of diagnostic markers such as the ones for *Rrs1* (Looseley et al. 2020) or *Rrs2* (Hanemann et al. 2009) is needed together with studies that quantify novel resistance sources from germplasm.

Genome-wide association studies (GWAS) can be used to detect QTL associated with interesting traits such as disease resistance (Alqudah et al. 2020). GWAS can be used in unrelated populations in contrast to QTL mapping where biparental populations are required (Cavanagh et al. 2008), and this enables to study wide allelic diversity (Rafalski 2010). A disadvantage of diversity panels compared to biparental populations is that the population structure becomes more complex, which may fade some of the allelic effects, and additionally, some rare alleles with low frequency may remain undetected (Huang and Han 2014).

Multi-parent Advanced Generation Inter-Cross (MAGIC) populations for GWAS combine the advantages of both biparental and unrelated populations (Huang et al. 2015; Scott et al. 2020). MAGIC populations typically consist of 4, 8 or 16 founders (parents), which are inter-crossed in separate groups for several generations, and then, individuals from different groups are inter-crossed. Subsequently recombinant inbred lines (RIL) or doubled haploids (DH) are produced (Huang et al. 2015; Scott et al. 2020). The limited number of founders increases the allele frequencies of MAGIC populations compared to unrelated populations, thus improving the detection of rare alleles (Scott et al. 2020). The inter-crossing of all founders with each other increases genetic variation, recombination and the number of polymorphisms compared to biparental crosses. Simultaneously, it reduces the decay of linkage disequilibrium (LD) compared to unrelated populations, but not when compared to biparental crosses. Additionally, novel allele combinations that are not present in the founder lines can be achieved through inter-crossing (Huang et al. 2015), and in the best-case scenario pyramiding of genes for traits of interest can be done while developing MAGIC populations, and no backcrossing is needed for this purpose (Scott et al. 2020). MAGIC populations have already been used in barley to study traits such as interaction of dwarfing genes and agronomic traits (Dang et al. 2020) or epistasis for flowering time (Mathew et al. 2018). Recently, powdery mildew resistance was successfully detected from the same four barley MAGIC populations that are studied here by Novakazi et al. (2020).

The single-locus model is the most commonly used GWAS model, but it does not take into account that there may be several QTL and this may reduce its statistical power and lead to biased effect estimates and increased Type I and Type II errors (Zhang et al. 2019). This has led to the development of several multi-locus models that may increase the power in QTL detection (Zhang et al. 2019).

The Nordic Pre-breeding collaborative (PPP barley consortium) has developed four Nordic spring barley MAGIC populations pyramiding resistance towards diseases such as scald, net blotch (*Pyrenophora teres* Drechsler 1923), Fusarium head blight (*Fusarium graminearum* Schwabe 1839), leaf rust (*Puccinia hordei* G.H. Otth 1871) and powdery mildew (*Blumeria graminis* (DC.) Speer 1975). These MAGIC populations were evaluated for scald resistance under field conditions in Finland and Iceland between 2017 and 2019. Here we report of three putatively new QTL located on chromosomes 1HS and 3HS and 5HL and allele combinations associated with scald resistance in Nordic spring barley, detected from MAGIC populations by using a multi-locus genome-wide association approach in *Genomic Association and Prediction Integrated Tool* (*GAPIT*).

## Material and methods

### Multi-Parent Advanced Generation Inter-Cross (MAGIC) populations

**Fig. 1:**
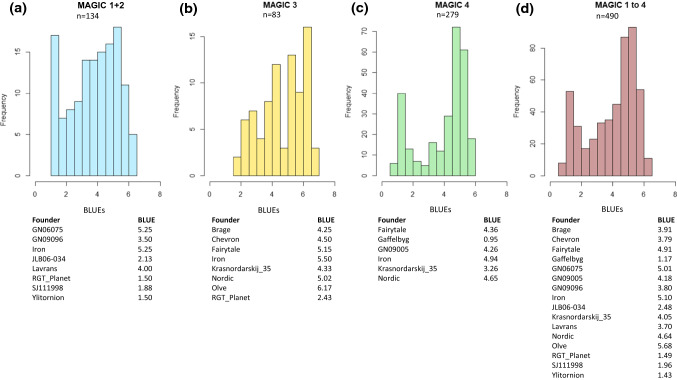
**a–d** Distribution of best linear unbiased estimators for scald scores (BLUEs) in separate MAGIC populations [MAGIC 1 + 2 in (**a**), MAGIC 3 in (**b**), MAGIC 4 in (**c**) and MAGIC 1 to 4 in (**d**)] studied and among the founders in each population

Four separate MAGIC populations were investigated in this study. The founders (Fig. 1) were selected based on their resistance against economically important diseases of barley (Bengtsson et al. 2017), but at least one agronomically adapted genotype was included as a founder. The development as well as structure of these populations is described in a study by Novakazi et al. (2020), which is on powdery mildew resistance within these populations. As mentioned by Novakazi et al. (2020), MAGIC 2 yielded only 29 lines. However, since seven of eight founders were the same for MAGIC 1 and MAGIC 2, these two populations were combined and considered as one population in further analyses. Analyses were performed separate for MAGIC 1 + 2, MAGIC 3 and MAGIC 4, and across populations (MAGIC 1 to 4).

### Field trials and phenotypic evaluation

MAGIC 1 + 2 was tested on the Natural Resources Institute Finland’s (Luke) experimental farm in Jokioinen Finland in 2018–2019 (Table 1). MAGIC 3 was tested in Jokioinen in 2017–2019 and on Korpa experimental farm in Reykjavik, Iceland, in 2017 and in Hvanneyri, Iceland (Agricultural university of Iceland) in 2018. MAGIC 4 was tested in Jokioinen in 2017–2019 and in addition in Luke’s experimental farm in Sotkamo Finland in 2019.

**Table 1:** Field evaluation details for assessment of scald resistance in four barley MAGIC populations

Location	Year	Sowing day	Replications	Observation day	MAGIC population(s) evaluated
Jokioinen	2017	5-June	2	7-August	4
Korpa	2017	31-May	2	27-July	3
Jokioinen	2018	21-May	2	23-July & 6-August	1 to 4
Hvanneyri	2018	8-June	2	13-August & 27-August	3
Jokioinen	2019	3-June	2	15-July & 5-August	1 to 4
Sotkamo	2019	20-May	1	6-August & 24-August	4

All field trials were set up in an alpha lattice design with two replications, except for Sotkamo 2019, which had only one replication. Disease severity was observed at two time-points at each location, except in Korpa and Jokioinen in 2017, where only one observation was performed due to low disease severity (Table 1).

Scald infection was based on natural infection in Sotkamo and on artificial inoculation in Jokioinen, Korpa and Hvanneyri. Inoculum was produced in the greenhouse by spraying a susceptible barley cultivar ‘Voitto’ with a mixture of *R. commune* isolates representing the natural variation in Finland and Iceland, respectively, at growth stage GS 12–13 (BBCH scale, Hack et al. (1992)) with a spore concentration of 10^6^ spores/mL. Two to three weeks after inoculation, plants were cut into pieces of 5 cm and dried at room temperature. The barley MAGIC lines were sown as hill plots with 20 seeds per plot. At GS 10, the infected dried leaves were spread between the rows. To ensure sufficient moisture and enhance disease development, trials were irrigated according to the need. Disease severity was assessed using a rating scale from 1–9, where 1 represents no infection and 9 dead plants with no green tissue left.

### Statistical analyses

Descriptive statistics were calculated for each population for all observations separately and combined, using the psych software package v. 1.8.12 (Revelle 2020) in R (R Core Team 2017). Box plot graphs and the pairwise Spearman’s rank correlations were calculated in SAS between each observation in each population using mean values from lines (SAS Enterprise guide v. 7.1) (Online Resource 1).

Statistical analyses and the best linear unbiased estimators (BLUEs) for scald were calculated across the environments for each population (Fig. 1), using the software META-R (Alvarado et al. 2020). Environments (year*location combinations) were considered random, whereas genotypes, replicates and genotype by environment interactions were considered as fixed effects. The BLUEs for each population and across populations were then used as phenotype values for the association mapping.

### Association mapping

The lines were genotyped with the 50 K Illumina Infinium iSelect array for barley. SNP filtering was performed as described in Novakazi et al. (2020). For GWAS, the SNPs were further filtered for Minor Allele Frequency (MAF) ≤ 0.05, resulting in 25,068 polymorphic SNPs for MAGIC 1 + 2, 18,103 SNPs for MAGIC 3, 19,072 SNPs for MAGIC 4 and 24,638 polymorphic SNPs for the combined populations MAGIC 1 to 4 to be used in the association mapping. The physical positions based on the barley reference genome, Morex 1.0, (Bayer et al. 2017; Mascher et al. 2017) were retrieved using the online tool BARLEYMAP (http://floresta.eead.csic.es/barleymap/) (Cantalapiedra et al. 2015). Population structure and Linkage disequilibrium were analysed in previously reported GWAS for mildew (Novakazi et al. 2020).

Five models were compared for the GWAS: General Linear Model (GLM), Mixed Linear Model (MLM) (Zhang et al. 2010), Multiple Loci Mixed linear Model (MLMM, Segura et al. 2012), Fixed and random model Circulating Probability Unification (FarmCPU, Liu et al. 2016) and Bayesian information and Linkage disequilibrium Iteratively Nested Keyway (BLINK, Huang et al. 2019) using the R package *GAPIT* (Lipka et al. 2012). In order to select the most suitable model covariate combination to account for population structure, the kinship matrix (K) calculated in *GAPIT* with the Van Raden method (Van Raden 2008), the ancestry coefficient data (Q matrix) obtained from STRUCTURE, calculated previously by Novakazi et al. (2020), and the principal component analysis (PCA) covariates from *GAPIT* were incorporated into the models and compared. For model comparison, the least deviation from the expected p-values was used as the primary criterion, and in addition highest number of groups, high -2 log likelihood value (-2LL) and lowest variance error were compared when available. When several models looked suitable, Manhattan plots, generated with the R package *CMPlot*, were observed and the model with most peaks associated with known resistance QTL for scald was selected. The Bonferroni thresholds for significant associations were calculated based on the number of effective markers (MAGIC 1 + 2 n = 4226, MAGIC 3 n = 1618, MAGIC 4 n = 1999, MAGIC 1 to 4 n = 4923) with α = 0.05 (Li et al. 2012).

Candidate genes, their locations and annotations were retrieved from the BARLEYMAP website (Cantalapiedra et al. 2015, http://floresta.eead.csic.es/barleymap/). The gene search around the peak markers was spread to match the genome-wide LD decay (determined in Novakazi et al. 2020) of the respective population studied. Haplotypes were formed based on the significant markers of each population separately and for the combined populations. The effect of each allele combination with at least five observations (lines) was calculated based on BLUE values, and the significance of the effects was tested using the linear model function in R.

### Diagnostic marker

DNA was extracted from homogenized freeze-dried leaf samples by a QIAcube HT extraction and the QIAamp 96 DNA QIAcube HT Kit (Qiagen, Hilden, Germany), as previously described in Åhman and Bengtsson (2019). DNA samples of Magic 1 to 4 were analysed for *Rrs2* with kompetitive allele-specific PCR (KASP) assays (LGC Genomics) following the manufacturers guidelines. Primers were designed by LGC Genomics based on sequence FJ974009 in Hanemann et al. 2009. Forward primers were CAAGGAGGTCCTTGGCCCC (susceptible allele), GTCAAGGAGGTCCTTGGCCCT (resistant allele), and common primer was GCACCTGAACGTCACCCAGGAA. The PCR contained 2.5 μl of DNA (30 ng/μl), 2.5 μl KASP Master mix (LGC Biosearch Technologies) and 0.07 μl of primer mix (12 μM of each allele-specific primer and 30 μM of reverse primer). Reaction was performed in Applied Biosystems™ QuantStudio™ 5 Real-Time PCR System, 384-well with the following cycling conditions: 15 min at 94 °C; 10 touchdown cycles of 20 s at 94 °C, 60 s at 61–55 °C (dropping 0.6 °C per cycle); and 41 cycles of 20 s at 94 °C, 60 s at 55 °C and 18 s at 35 °C. Fluorescence reading was taken at 40 °C for 30 s and analysed using cloud-based Thermo Fisher Connect Genotyping.

## Results

### Phenotypic evaluation

All data concerning analysis of phenotypic data are found in Fig. 1 and in Online Resource 1. The disease severity in the second observation at GS 75 was usually higher than the first, except for Iceland, where this could be due to different individuals conducting the screening. Significant correlations (p ≤ 0.001) were found between all observations per population. The observations between Iceland and Finland showed the lowest correlation with r > 0.47 (Online Resource 1).

The frequency distributions of all populations were skewed with higher proportion of low disease scores except the first observation in Sotkamo for MAGIC 4 which was right skewed (Fig. 1). Statistical analysis revealed that in all populations, effect of the genotype was highly significant, but the effect of environment was only significant for MAGIC 3. G × E interaction had significant impact on the disease scores in all populations except MAGIC 4 (Online Resource 1). The broad sense heritability was high for all populations studied and ranged from H^2^ = 0.78 in MAGIC 1 + 2 to 0.91 in MAGIC 4 (Online Resource 1), indicating large influence of genetic variation to observed phenotypic variation.

Differences between founders of MAGIC populations were distinguishable (Fig. 1). There were highly resistant genotypes that had BLUEs below 2 including ‘Gaffelbyg’, ‘SJ111998’, ‘RGT Planet’ and estimates as high as 5 and above on founders ‘GN06075’, ‘Iron’ and ‘Olve’. Founder MBR-1012, which was not included in GWAS due to low call rate (Novakazi et al. 2020), had an above average disease score of 4.53 ± 1.33 (Mean ± SD) based on 13 field observations within the trials of MAGIC 1 + 2.

### Model selection

**Fig. 2:**
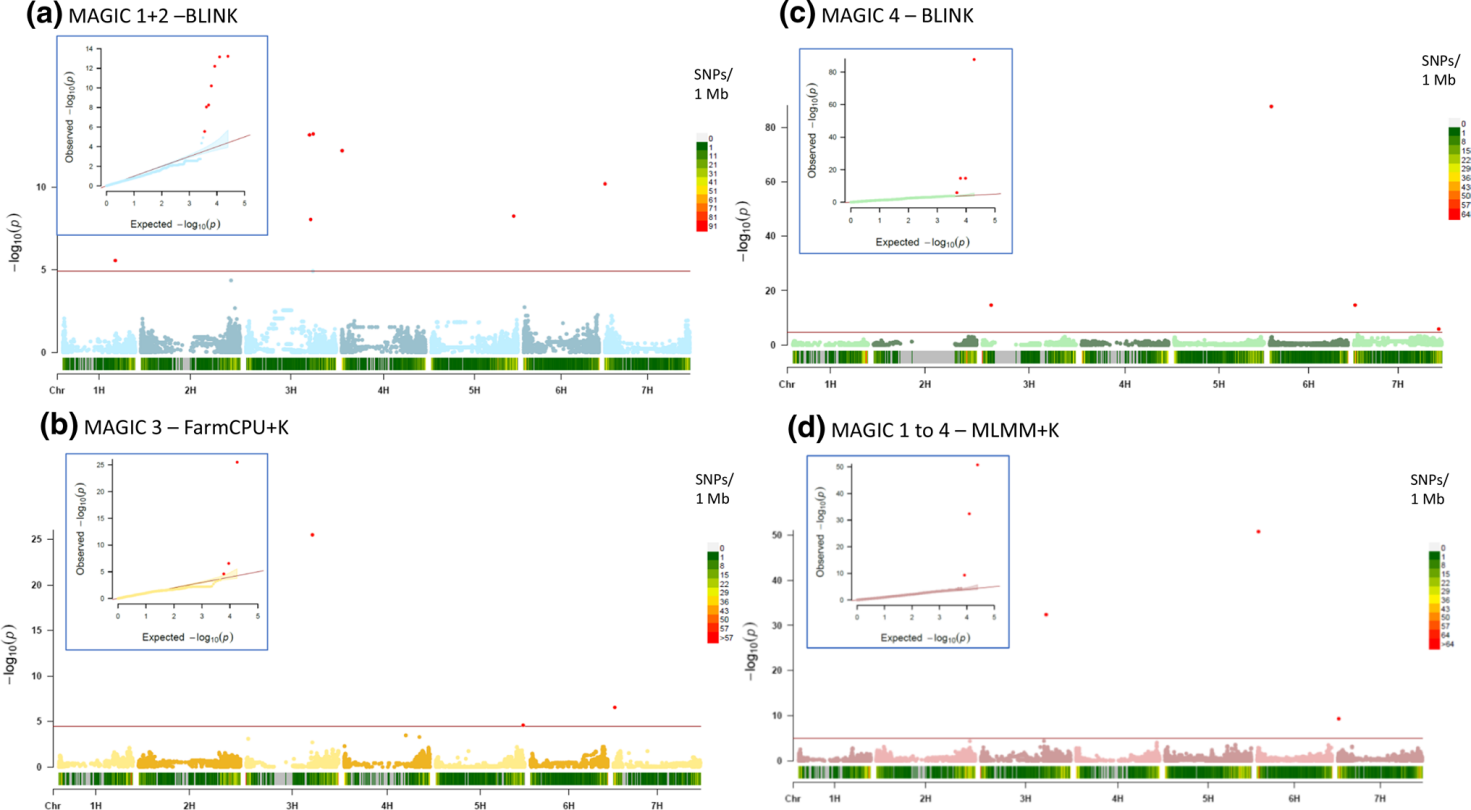
**a-d** Genome-wide association analysis for leaf scald resistance in four barley MAGIC populations MAGIC 1 + 2 (**a**, light blue), MAGIC 3 (**b**, golden), MAGIC 4 (**c**, green) and MAGIC 1 to 4 (**d**, rosybrown). Manhattan plots of the best model and respective quantile–quantile (QQ) plots for each population are displayed. The horizontal axis shows the seven barley chromosomes with physical positions in bp, the vertical axis presents the –log10 (*p*)-values. The *red horizontal line* represents the Bonferroni adjusted significance threshold –log10 (*p*) with values of 4.93 (MAGIC 1 + 2), 5.1 (MAGIC 3), 4.6 (MAGIC 4), and 4.99 (MAGIC 1 to 4), corresponding to an error rate of 0.05

Based on the Bayesian information criterion (BIC) and maximum log likelihood values, implemented in the model selection option in *GAPIT*, a principal component was included in the GWAS for population MAGIC 4 but not for MAGIC 1 + 2, MAGIC 3 or MAGIC 1 to 4. Based on the model selection criteria, BLINK was the best model for MAGIC 1 + 2 and MAGIC 4, whereas FarmCPU + kinship (K) was the preferred model for MAGIC 3 (Fig. 2, Online Resource 2). The best model for the combined populations (MAGIC 1 to 4) was MLMM + K (Fig. 2, Online Resource 2).

### Marker trait associations (MTAs)

Logarithm of odds (LOD) thresholds for MAGIC 1 + 2, MAGIC 3, MAGIC 4 and MAGIC 1 to 4 were 4.93, 4.51, 4.6 and 4.99, respectively. A total of 17 MTAs were detected, corresponding to nine distinct loci, located on chromosomes 1H, 3H, 4H, 5H, 6H and 7H (Table 2, Fig. 2, Online Resource 3). MAGIC 1 + 2 had six distinct QTL, MAGIC 3 had three, MAGIC 4 had four, and the three significant markers associated in MAGIC 1 to 4 co-localised with three separate QTL detected in the individual populations.

**Table 2:** QTL for scald resistance identified in genome-wide association studies in four barley MAGIC populations, markers significantly associated with these regions and their physical locations (Morex 1.0, Mascher et al. 2017), as well as founders and lines carrying the favourable alleles

QTL	SNP	Chromosome	Position [bp]	*p-*value	LOD	MAF	Effect	MAGIC Population(s)	Founder with favourable allele	Lines with favourable allele (including founders)
Qsc_1H_1	JHI_Hv50k_2016_29000	1H	404,905,055	2.75E−06	5.56	0.25	0.34	1–2	RGTPlanet, SJ111998, Ylitornion, Iron, JBL06-034, MBR-1012	102
Qsc_3H_1	JHI_Hv50k_2016_164742	3H	63,225,476	2.04E−15	14.69	0.44	0.94	4	Gaffelbyg, Iron, Krasnodarskij35	157
Qsc_3H_2	JHI_Hv50k_2016_183433	3H	491,084,467	6.83E−14	13.17	0.50	0.65	1–2	SJ111998, RGTPlanet, Ylitornion, Lavrans	67
	SCRI_RS_168665	3H	499,413,118	3.17E−26	25.50	0.31	− 1.24	3	RGTPlanet	26
				8.96E−09	8.05	0.28	− 1.02	1–2	RGTPlanet	38
	JHI_Hv50k_2016_185295	3H	507,319,913	4.25E−33	32.40	0.10	− 0.86	1–4	RGTPlanet, GN09005	48
Qsc_3H_3	JHI_Hv50k_2016_186622	3H	519,522,630	6.00E−14	13.22	0.16	0.75	1–2	RGTPlanet, GN06075, GN09096, JLB06-034, Lavrans, SJ111998, Ylitornion	113
Qsc_4H_1	JHI_Hv50k_2016_226785	4H	1,346,098	6.19E−13	12.21	0.34	− 0.82	1–2	RGTPlanet, SJ111998, Ylitornion, JLB06-034, MBR-1012	46
Qsc_5H_1	JHI_Hv50k_2016_350540	5H	638,245,248	5.56E−09	8.25	0.48	− 0.76	1–2	Iron, RGTPlanet, SJ111998, Ylitornion, JLB06-034, MBR-1012	64
	BOPA2_12_30577	5H	659,532,782	2.51E−05	4.60	0.45	− 0.26	3	Iron, RGTPlanet, Chevron, Fairytale, Nordic	37
Qsc_6H_1	JHI_Hv50k_2016_373110	6H	10,924,478	1.76E−51	50.80	0.16	− 1.36	1–4	Gaffelbyg, JLB06-034, SJ111998	80
				2.03E−88	87.69	0.24	− 1.57	4	Gaffelbyg	66
Qsc_7H_1	JHI_Hv50k_2016_436076	7H	787,436	6.25E−11	10.20	0.09	− 0.32	1–2	Lavrans, GN09096	12
	JHI_Hv50k_2016_442495	7H	6,808,999	4.68E−10	9.33	0.17	− 0.79	1–4	Brage, Lavrans, GN09096, GN09005	81
	SCRI_RS_42792	7H	8,729,097	2.01E−15	14.70	0.20	− 1.08	4	GN09005	56
				2.82E−07	6.55	0.27	− 0.40	3	Brage, Chevron	22
Qsc_7H_2	SCRI_RS_193330	7H	635,235,975	1.34E−06	5.87	0.46	0.20	4	Fairytale, Nordic, Krasnodarskij35	150

Complete lists can be found in Online Resource 4 and 5. Effects are calculated with “Major.allele.zero = TRUE” setting in *GAPIT*; thus, the sign of the allelic effect estimate is with respect to the minor allele

The first QTL, Qsc_1H_1, was located on chromosome 1H at 404.9 Mb and was detected only in MAGIC 1 + 2 with the peak marker (JHI_Hv50k_2016_29000) showing a LOD value of 5.56 (Table 2). On chromosome 3H, three QTL were detected. The first, Qsc_3H_1, was detected in MAGIC 4 with the peak marker (JHI_Hv50k_2016_164742) at 63.2 Mb and relatively high LOD value of 14.69. The second QTL was located between 491 and 507.3 Mb with LOD values ranging from 8.05 (SCRI_RS_168665 in MAGIC 1 + 2) to 32.40 (same marker in MAGIC 3). This QTL, Qsc_3H_2, was associated with three separate markers in all populations except MAGIC 4. In MAGIC 1 + 2, also a separate QTL, Qsc_3H_3, was detected close to Qsc_3H_2 at 519.5 Mb (JHI_Hv50k_2016_186622) with a LOD value of 13.2.

One significant marker (JHI_Hv50k_2016_226785) was detected on chromosome 4H with LOD value of 12.21 in MAGIC 1 + 2. On chromosome 5H, a QTL, Qsc_5H_1, between significant markers at 638.2 and 659.5 Mb was detected in MAGIC 1 + 2 and MAGIC 3 with LOD values between 4.60 (MAGIC 3) and 8.25 (MAGIC 1 + 2) (Table 2). The peak marker JHI_Hv50k_2016_373110 on chromosome 6H at 10.9 Mb was significant in MAGIC 4 and MAGIC 1 to 4 and had the highest LOD values of 50.80 and 87.69 within this study (Table 2).

On chromosome 7H, two QTL were detected (Table 2). The first, Qsc_7H_1, can be found in all populations. The peak markers JHI_Hv50k_2016_442495, JHI_Hv50k_2016_436076, and SCRI_RS_42792 were located between 0.7 Mb (LOD 10.20) and 8.7 Mb (LODs 14.70). The last region, Qsc_7H_2, was identified only in MAGIC 4 at 635.2 Mb with a LOD of 5.9 for the peak marker SCRI_RS_193330.

### Candidate genes

**Fig. 3:**
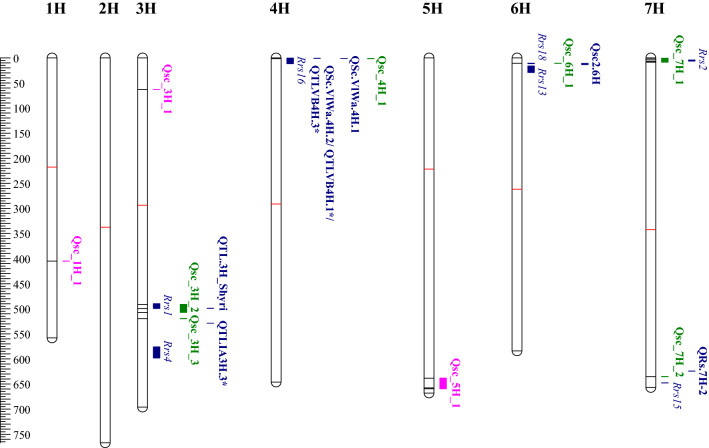
Detected QTL for scald in the present study and closely located QTL reported in literature. Novel QTL are indicated with pink colour, green colour indicates other QTL found in this study and blue QTL are reported in literature. The red line is the centromere (Mascher et al. 2017) and the black lines are the positions of the significant SNPs. Positions are retrieved from original publications or if the original position was not applicable to Morex 1.0. the marker information was retrieved from Zhang et al. (2020) and repositioned to Morex 1.0 with the help of Grain Genes database (https://wheat.pw.usda.gov/GG3/). *indicates that the name of the QTL comes from a review by Zhang et al. 2020

All QTL contained genes that can be associated with plant defence. In most of the regions, at least one and often several disease resistance proteins and leucine-rich repeats (LRR) were located. Genes involved in pathogen recognition and signal transduction such as PRR-receptors, serine–threonine protein kinases and genes encoding responsive proteins such as peroxidases, pectinases, chitinases, cellulose and callose synthases are found around the significant markers (Online Resource 4). Literature reports several known resistance genes that are likely to work behind the QTL detected on chromosomes: 3H, 4H, 6H and 7H (Zhang et al. 2020) (Fig. 3).

### Allele combinations

**Fig. 4:**
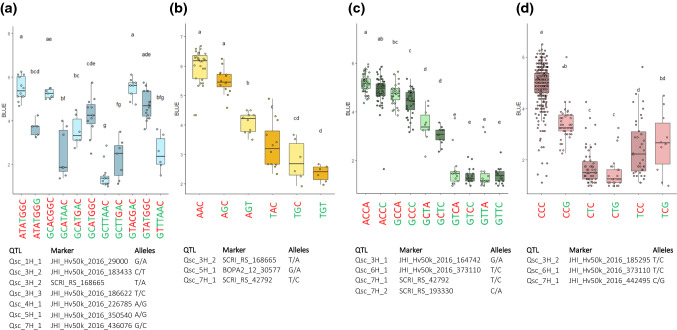
**a-d** Boxplot of allele combinations showing BLUEs for Scald scores in the four MAGIC populations: MAGIC 1 + 2 (**a**), MAGIC 3 (**b**), MAGIC 4 (**c**) and combination of all populations (**d**). The effect of each allele combination for combinations with at least five observations (doubled haploid lines or founders) was calculated based on BLUE values. Allele combinations followed by the same lower-case letter do not differ statistically. Favourable alleles are indicated with green and unfavourable with red colour on the haplotype names under the figures

In order to find lines with favourable allele combinations, haplotypes were formed based on the significant markers for each population. Table 2 shows which founder contributed the favourable allele for each QTL and how many lines carry this allele. Haplotype formation for MAGIC 1 + 2 revealed 34 different haplotypes (Online Resource 5). None of the lines or founders in MAGIC 1 + 2 had all favourable alleles, but combinations of six favourable alleles were detected in 15 genotypes, and these had average disease scores below 2, whereas no favourable alleles or only one favourable allele led to average disease scores above 5 (Fig. 4a).

Three significant SNPs of MAGIC 3 combined into eight haplotypes (Online Resource 5) with average BLUEs ranging from 2.35 to 5.96. Six lines contained all three favourable alleles and had the lowest disease scores among the haplotypes (Fig. 4b). Combination of favourable alleles at Qsc_3H_2 and Qsc_5H_1 did not differ significantly from combination of all three favourable alleles, although the average score estimate for lines containing all three alleles was lower. Also, the additive effect can be demonstrated by the lines containing both favourable alleles for Qsc_7H_1 and Qsc_5H_1 having significantly lower disease estimates than lines containing only Qsc_5H_1.

MAGIC 4 had four significant SNPs and among these 13 different haplotypes were identified with average disease scores ranging from 1.37 to 5.17 (Fig. 4c, Online Resource 5). Four allele combinations differed from others by their low disease score (Fig. 4) but did not differ significantly from each other (scores between 1.37 and 1.63). All of these lines contained the favourable alleles from Qsc_3H_1 and Qsc_6H_1. Considering that the 31 lines containing only the favourable allele Qsc_3H_1 inherited from ‘Iron’ did not have big difference in their disease score (4.71) to the 57 lines without any favourable alleles (5.17), the Qsc_6H_1 is the major QTL in MAGIC 4. ‘Gaffelbyg’ was the only founder having the favourable allele from Qsc_6H_1, which is reflected in its low disease scores (Fig. 1) and it inherited it to 63 lines (Table 2). However, in MAGIC 1 to 4 this allele was also detected in two other founders, JLB06-034 and SJ111998, and in 12 lines in MAGIC 2.

MAGIC 1 to 4 had three significant SNPs and these resulted in six different haplotypes (Fig. 4d). No line combined all three favourable alleles, which indicates that these alleles originate from founders that are not common in all four MAGIC populations. All three QTL have a clear impact on disease reduction. The strongest reduction in disease scores results from Qsc_6H_1. Fifty-four genotypes had only the favourable allele from Qsc_6H_1 and showed low average BLUE values of 1.74. The lines carrying the QTL on chromosomes 3H and 7H have average BLUEs of 2.47 and 3.42, respectively (Online Resource 5).

464 out of 490 lines including all founders except ‘Iron’ were successfully diagnosed with a marker for *Rrs2* (Hanemann et al. 2009). In detail, 17.1, 29.5 and 16.7% of the diagnosed lines in MAGIC 1 + 2, MAGIC 3 and MAGIC 4, respectively, carried the *Rrs2* allele, as well as the founders: ‘Brage’, ‘GN09005’, ‘GN09096’, ‘Lavrans’ and ‘Ylitornion’.

## Discussion

### Known scald resistance QTL detected among the populations

The high proportion of lines with low disease severity in the phenotypic data already indicates that the studied populations harbour scald resistance within them. Many of the known resistance QTL are located on chromosomes 3H and 7H (Zhang et al. 2020), which also showed most associations in the present study. However, the QTL on chromosome 6H, Qsc_6H_1, had the most significant impact on scald resistance in the combined populations as well as in MAGIC 4 where it was identified.

The Danish landrace ‘Gaffelbyg’ was the one to contribute Qsc_6H_1 to MAGIC 4. Also, the Jordanian landrace ‘JLB06-034’ and Danish breeding line ‘SJ111998’ carried this SNP, but significant effect was not detected in MAGIC 1 + 2. The marker for Qsc_6H_1 corresponds directly to the proposed location of *Rrs18* located between 10.9 and 11.6 Mb (Coulter et al. 2019). However, chromosome 6H contains another major resistance QTL close to Qsc_6H_1/ *Rrs18*, designated *Rrs13* (Abbott et al. 1995) between 16 and 29.1 Mb (Cheong et al. 2006). Several other QTL in this region are identified (Zhang et al. 2020), and especially, the recently described QTL, Qsc2.6H, explaining over 70% of variation in a Canadian RIL population (flanking markers at 11.2 and 13.2 Mb, Zantinge et al. 2019) could be a relevant candidate for the QTL found in this study. Since *Rrs13* is a common gene among two-row spring barley germplasm (Looseley et al. 2018), it is possible that it has been introduced from some of the founder genotypes.

The first scald resistance locus discovered in barley, *Rrs1*, is mapped between 489,991,522 and 491,895,585 bp (Hofmann et al. 2013), and the significant marker for Qsc_3H_2, JHI_Hv50k_2016_183433 (491,084,467 bp), detected in MAGIC 1 + 2 lies within this region. The marker for the *Rrs1* (Rh4) allele is located at 490,253,069 (Looseley et al. 2020). The significant markers within MAGIC 3 and MAGIC 1 to 4 are also very close to this region and clearly within the range of linkage disequilibrium. The marker identified for another allele of *Rrs1* (QTL.3H‐Shyri, Zantinge et al. 2019) located at 498,944,660 bp is in close proximity to the significant marker SCRI_RS_168665 found at 499,413,118 from MAGIC 3 and MAGIC1 + 2, which was inherited from ‘RGT Planet’ in our study.

There are more than 11 alleles identified within this locus (Bjørnstad et al. 2002), but it is still unclear whether *Rrs1* represents several R-genes connected to each other or whether these are only alleles of the same locus, since twenty-seven major and minor QTL are identified between 470.5 and 554.5 Mb on chromosome 3H (between 428.8 and 503.4 Mb as reported by the most recent Morex assembly, Morex 2.0, Zhang et al. 2020). Only a few interesting genes were annotated close to the predicted location of *Rrs1,* but this is not a surprise since the variant behind this is regarded to be absent from the current Morex genome sequence (Looseley et al. 2020). The third marker associated with Qsc_3H_2 was found from MAGIC 1 to 4, and it is located at 507.3 Mb. Since it was found in the combined populations, this may explain the close existence of another QTL, Qsc_3H_3, which is located at 519.5 Mb. The closest QTL was reported by Grønnerød et al. (2002), and in Morex 1.0 this is located approximately between 528.3 and 528.6 Mb (the closest proteins matching with 480.5 Mb in Morex 2.0). Considering the number of QTL in this region, it is very likely that Qsc_3H_2 is either one of these QTL or a completely new one. The closest disease resistance genes “*HORVU3Hr1G068430*” and “*HORVU3Hr1G068410*” were located less than 1 Mb from the significant marker JHI_Hv50k_2016_186622. A less likely candidate for this QTL is *Rrs4,* which is mapped between 576 and 598 Mb (at 523.0 Mb on Morex 2.0) (Patil et al. 2003). An evasion mechanism that may influence results is maturation time (Zhang et al. 2019). When scald resistance was modelled together with relative maturity, a novel major QTL was identified on chromosome 3H (503.4 Mb, Morex 2.0, Zhang et al. 2019). Supporting our GWAS results where Qsc_3H_1 had fairly high impact on disease resistance, *Rrs1* was the QTL with most significant effect on scald resistance in a GWAS study performed on field conditions and for adult plants in European spring barley germplasm (Looseley et al. 2018). Additionally, it was found to be very common in UK spring barley germplasm and represented in over 50% of cultivars released after 2010 (Looseley et al. 2018)*.*

The QTL regions found in this study on chromosome 7H contain multiple disease resistance genes. Results from diagnostic markers strongly suggest that Qcs_7H_1 located on the short arm of chromosome 7H is *Rrs2* (Hanemann et al. 2009). All lines from the MAGIC 1 to 4 panel showing the favourable allele for the significant SNP JHI_Hv50k_2016_442495 were also diagnosed to carry *Rrs2*. However, the diagnostic marker detected more genotypes with *Rrs2* from the populations than the GWAS, since only 96.9% of genotypes diagnosed for *Rrs2* (n = 464) had the favourable allele for Qsc_7H_1, indicating that the already existing marker is probably more tightly linked to *Rrs2* than our SNP. For example, the founder ‘Ylitornion’ did not have the favourable allele at Qsc_7H_1 despite the marker associated with *Rrs2* being significant. *Rrs2* is effective against many isolates of *R. commune* and widely used in barley breeding programmes around the world (Hanemann et al. 2009). Interestingly, Looseley et al. (2018) did not detect *Rrs2* in a GWAS study based on screenings of 660 spring barley lines in disease nursery conditions, but they identified a nearby QTL based on an analysis with historical data collated from value for cultivation and use trials. That QTL was located in the same region as Qcs_7H_1 but did not match with *Rrs2* when compared with R-gene specific isolates. The other QTL detected in MAGIC 4 in our study had its peak marker on the long arm of chromosome 7H at 635.2 Mb, which is close to *Rrs15* located at 647.6 Mb (Genger et al. 2003). An even closer QTL is the recently reported new QTL QRs7H.2 624.2 Mb, which was found by Büttner et al. (2020) in a barley nested association mapping population, HEB-25 (Maurer et al. 2015). QTL close to the significant marker can be considered as very relevant candidates. The recurrent parent in HEB-25 is ‘Barke’, which was crossed with 25 wild barley (*H. spontaneum*) accessions from the fertile crescent and thus having clearly higher amount of diversity introduced in their material compared to ours. Interestingly, out of the eight QTL Büttner et al. (2020) found two others in addition to QRs7H.2, which are potentially the same as ours: the one considered as *Rrs1* on chromosome 3H and possibly the QTL on 6H at 18.9Mbp.

Chromosome 4H harbours one significant marker at 1.3 Mb in MAGIC 1 + 2. The closest major resistance locus is *Rrs16* introgressed from *Hordeum bulbosum* (Pickering et al. 2006) and is mapped between 0.6 and 11.7 Mb. There are also other QTL with major effects close to the *Rrs16* locus suggesting that this region harbours resistance (Wallwork et al. 2014; Wang et al. 2014). These two studies traced the resistance back to cultivar ‘Vlaming’ that was released in 2006. However, the present study found this resistance locus also in the landraces ‘Ylitornion’ and ‘JBL06-034’ which suggests that *Rrs16* is not only present in *H. bulbosum*.

### Three putatively new resistance QTL

Three putatively novel QTL are identified in this study: Qsc_1H_1, Qsc_3H_1 and Qsc_5H_1. Qsc_3H_1 lies within the gene HORVU3Hr1G020310 at 63.2 Mb (at 53.2 Mb at Morex 2.0). There is a minor QTL, QSc.TxFr-3H, reported at 39.3 Mb (32.3 Mb in Morex 2.0) by Li and Zhou (2011), but even this is over 20 Mb from our detected marker, indicating that this is a putatively new resistance gene. *HORVU3Hr1G020310* is a homeodomain-like transcriptional regulator, and it may well have a role in disease resistance (Coego et al. 2005). Less than one Mb downstream from the marker, JHI_Hv50k_2016_164742, is a FAD-binding Berberin family protein that could inhabit antifungal properties (Freile et al. 2006). Additionally, several heat shock proteins lie very close to the marker for Qsc_3H_1 (Online Resource 4) and these can interact with resistance proteins (De La Fuente Bentem van et al. 2005).

Chromosomes 1H and 5H harbour only a few reported scald resistance QTL (Zhang et al. 2020). The marker at Qsc_1H_1 was situated at a gene with unknown function that was right next to a metacaspase protein-encoding gene (*HORVU1Hr1G055210*, Online Resource 4) which is known to execute hypersensitive reaction in plants (Gong et al. 2019). Ten disease resistance proteins were detected between the flanking markers at Qsc_5H_1. In addition, the region contained multiple leucine-rich domains and calmodulin proteins related to stress–response calcium signalling (Reddy et al. 2011) (Online Resource 4).

### Resistance pyramided for scald

The founder ‘RGT Planet’ points out as moderately resistant in both populations, MAGIC 1 + 2 and MAGIC 3 (Fig. 1). ‘RGT Planet’ has six out of seven favourable alleles (haplotype GCTTAAC in Fig. 4a) detected in MAGIC 1 + 2 and two out of three favourable alleles in MAGIC 3. However, when the haplotypes of MAGIC 1 to 4 were studied, this cultivar did not carry the favourable alleles associated with the significant markers located on chromosome 6H and 7H. This indicates that crossing haplotypes containing the favourable alleles found in ‘RGT Planet’ with the 26 MAGIC 4 lines with a haplotype containing the favourable alleles from 6 and 7H can further improve resistance through pyramiding alleles. Overall, these MAGIC populations successfully enriched resistance genes in breeding material. Thirteen lines with the same haplotype as ‘RGT Planet’ were detected from MAGIC 1 + 2. In addition, one haplotype of five favourable alleles but with one favourable allele that was not present in ‘RGT Planet’ was detected in MAGIC 1 + 2. MAGIC 3 and MAGIC 4 generated six and 17 lines, respectively, that contained all favourable alleles detected in the corresponding MAGIC population and thus outperformed their founders.

### Pyramiding resistance for scald and powdery mildew

Seven lines and ‘RGT Planet’ out of the 14 genotypes with five favourable alleles increasing scald resistance in MAGIC 1 + 2 also carried three out of four alleles detected to increase mildew resistance by Novakazi et al. (2020). Two of the lines carrying all four favourable alleles associated with reduced mildew severity in MAGIC 1 + 2 also contained five favourable scald alleles. Similarly, in MAGIC 3, one of the six lines containing all of the favourable alleles in MAGIC 3 also contained four out of five favourable alleles for mildew and two other of these lines contained three favourable mildew alleles. Lines with promising gene combinations against a hemibiotrophic and a biotrophic pathogen are not something that can be taken for granted. Powdery mildew is an obligate biotroph (Liang et al. 2018), and *R. commune* is classified as a hemibiotroph, since it produces necrotic lesions after a long asymptomatic phase where it has been shown to colonize the host and sporulate (Avrova and Knogge 2012). McGrann et al. (2014) reported from a negative trade-off between *mlo*-resistance and resistance to Ramularia leaf spot disease in barley. Our results do not indicate that this kind of negative relationship exists between mildew and scald resistance in the studied germplasm, since there was no correlation between the scald estimates and mildew estimates, and also the founders ‘RGT Planet’ and ‘SJ111998’ both contained the *mlo-11* allele, but were also resistant to scald.

### Variable germplasm in highly infectious environment

The genetic architecture of scald resistance is complex, and the effects of resistance genes vary from one barley-growing area to the other (Zhang et al. 2020). Our study was conducted in two Nordic countries, Finland and Iceland. These countries are likely to differ from Central Europe regarding the virulence structure of *R. commune* populations. However, McDonald (2015) concluded that Scandinavia, which is the predicted origin of the pathogen (Brunner et al. 2007), is the best location to assess the durability of new sources of scald resistance because it has the most diverse *R. commune* populations that are likely to carry the greatest diversity for virulence.

MAGIC 3 that was screened for scald both in Iceland and Finland was also compared by separate GWAS between these countries to see if there are isolate specific associations. But no country-specific QTL were detected (data not shown) as could be assumed from the high correlations between the observations. Both in Iceland and in Finland, a mixture of local *R. commune* isolates was applied. Moreover, MAGIC 4, was evaluated under natural infection in Sotkamo, Finland, where high disease scores are expected from year to year. Even though there are no further studies available regarding the virulence of the individual isolates at different locations, we can predict that the resistance found in this study had to be effective against more than one pathotype of *R. commune* in order to contribute low disease scores across several locations.

Landraces have been sources of effective resistance for decades, and they still are a considerable option for resistance breeding (van Leur et al. 1989; Piechota et al. 2019; Looseley et al. 2020). A challenge in the use of landraces is that they also harbour many undesirable traits. Our study has shown examples of landraces such as ‘Gaffelbyg’, contributing *Rrs18,* and ‘Ylitornion’, which possibly had the same resistance gene that was previously found only in *H. bulbosum*. Landraces can still contain unused potential for disease resistance breeding, and by using MAGIC populations, these traits can be brought closer to modern cultivars. However, much of the resistance that is gathered in the MAGIC populations studied here is a result from decades of resistance breeding, especially in Norway where the founders: ‘Lavrans’, ‘Brage’, ‘GN06075’, ‘GN09005’ and ‘GN09096’ have been developed. The work dates way back to the utilization of genomic markers; thus, pyramiding of resistance in the breeding material was confirmed by using several strains of *R. commune* with different virulence genes (L. Reitan, personal communication).

## Conclusions

Nordic collaboration in pre-breeding through joint research, and especially phenotyping efforts, is a powerful strategy to enhance disease resistance, since more material can be studied in different climatic conditions than would have been possible to an individual company or research organization. Valuable combinations of known and new resistance QTL against scald in spring barley were detected from four MAGIC populations when models MLMM, FarmCPU and BLINK, all considering existence of multiple QTL, were applied in GWAS. The identified QTL and genotypes with pyramided resistance will be applied in the future breeding of spring barley in Northern Europe. Future studies in the same populations infected with leaf rust and Fusarium head blight may further enlighten how resistance genes against several diseases can be pyramided through MAGIC populations.

## Supplementary Information

Below is the link to the electronic supplementary material.

Supplementary material 1 (PDF 175 kb)

Supplementary material 2 (PDF 2,519 kb)

Supplementary material 3 (XLSX 4,598 kb)

Supplementary material 4 (XLSX 436 kb)

Supplementary material 5 (XLSX 2,912 kb)

## Data Availability

GWAS results are provided as supplementary material.

## Code availability

Not applicable, software used according to manuals.

## References

[CR1] Abang MM, Baum M, Ceccarelli S, Grando S, Linde CC, Yahyaoui A, Zhan J, McDonald BA (2006). Differential selection on *Rhynchosporium* secalis during parasitic and saprophytic phases in the barley scald disease cycle. Phytopathology.

[CR2] Abbott DC, Lagudah ES, Brown AHD (1995). Identification of RFLPs flanking a scald resistance gene on barley chromosome 6. J Hered.

[CR3] Arvidsson J (1998). Effects of cultivation depth in reduced tillage on soil physical properties, crop yield and plant pathogens. Eur J Agr.

[CR72] Åhman I, Bengtsson T (2019). Introgression of resistance to *Rhopalosiphum padi* L. from wild barley into cultivated barley facilitated by doubled haploid and molecular marker techniques. Theor Appl Genet.

[CR4] Alqudah AM, Sallam A, Stephen Baenziger P, Börner A (2020). GWAS: fast-forwarding gene identification and characterization in temperate Cereals: lessons from Barley–a review. J Adv Res.

[CR5] Alvarado G, Rodríguez FM, Pacheco A, Burgueño J, Crossa J, Vargas M, Pérez-Rodríguez P, Lopez-Cruz MA (2020). META-R: a software to analyze data from multi-environment plant breeding trials. Crop J.

[CR6] Avrova A, Knogge W (2012). *Rhynchosporium commune*: a persistent threat to barley cultivation. Mol Plant Pathol.

[CR7] Bayer MM, Rapazote-Flores P, Ganal M (2017). Development and evaluation of a barley 50k iSelect SNP array. Front Plant Sci.

[CR8] Bengtsson T, Åhman I, Manninen O (2017). A novel QTL for powdery mildew resistance in nordic spring barley (*Hordeum vulgare* L. ssp. vulgare) revealed by genome-wide association study. Front Plant Sci.

[CR9] Bjørnstad Å, Patil V, Tekauz A, Marøy AG, Skinnes H, Jensen A, Magnus H, MacKey J (2002). Resistance to scald (*Rhynchosporium secalis*) in barley (Hordeum vulgare) studied by near-isogenic lines: I. Markers and differential isolates. Phytopathology.

[CR10] Brunner PC, Schurch S, McDonald BA (2007). The origin and colonization history of the barley scald pathogen *Rhynchosporium secalis*. J Evolution Biol.

[CR11] Büttner B, Draba V, Pillen K (2020). Identification of QTLs conferring resistance to scald (*Rhynchosporium commune*) in the barley nested association mapping population HEB-25. BMC Genom.

[CR12] Cantalapiedra CP, Boudiar R, Casas AM (2015). BARLEYMAP: physical and genetic mapping of nucleotide sequences and annotation of surrounding loci in barley. Mol Breed.

[CR13] Cavanagh CR, Morell M, Mackay I, Powell W (2008). From mutations to MAGIC: resources for gene discovery, validation and delivery in crop plants. Curr Opin Plant Biol.

[CR14] Cheong J, Williams K, Wallwork H (2006). The identification of QTLs for adult plant resistance to leaf scald in barley. Aust J Agric Res.

[CR15] Coego A, Ramirez V, Gil MJ, Flors V, Mauch-Mani B, Vera P (2005). An Arabidopsis homeodomain transcription factor, Overexpressor of Cationic Peroxidase 3, mediates resistance to infection by necrotrophic pathogens. Plant Cell.

[CR16] Coulter M, Büttner B, Hofmann K (2019). Characterisation of barley resistance to *Rhynchosporium* on chromosome 6HS. Theor Appl Genet.

[CR17] Crous PW, Braun U, McDonald BA et al (2021) Redefining genera of cereal pathogens: *Oculimacula*, *Rhynchosporium* and *Spermospora*. Fungal Syst Evol 10.3114/fuse.2021.07.04 10.3114/fuse.2021.07.04 PMC8165968 34124618

[CR18] Dang VH, Hill CB, Zhang XQ (2020). Genetic dissection of the interactions between semi-dwarfing genes sdw1 and ari-e and their effects on agronomic traits in a barley MAGIC population. Mol Breeding.

[CR19] Davis H, Fitt BDL (1992). Seasonal changes in primary and secondary inoculum during epidemics of leaf blotch (*Rhynchosporium secalis*) on winter barley. Ann Appl Biol.

[CR20] de la Fuente van Bentem S, Vossen JH, de Vries KJ, van Wees S, Tameling WI, Dekker HL, de Koster CG, Haring MA, Takken FL, Cornelissen BJ. (2005) Heat shock protein 90 and its co-chaperone protein phosphatase 5 interact with distinct regions of the tomato I-2 disease resistance protein. Plant J 43:284–298. 10.1111/j.1365-313X.2005.02450.x 10.1111/j.1365-313X.2005.02450.x 15998314

[CR21] FAOSTAT (2020) Crops. http://www.fao.org/faostat/en/#data/SC Rome, Italy: Database of food and agriculture, Organization of the United Nations. Accessed 1 April 2021.

[CR22] Fitt BDL, Creighton NF, Lacey ME, McCartney HA (1986). Effects of rainfall intensity and duration on dispersal of *Rhynchosporium secalis* conidia from infected barley leaves. T Br Mycol Soc.

[CR23] Freile M, Giannini F, Sortino M, Zamora M, Juarez A, Zacchino S (2006). Antifungal activity of aqueous extracts and of berberine isolated from Berberis heterophylla. Acta Farm Bonaer.

[CR24] Genger RK, Williams KJ, Raman H, Read BJ, Wallwork H, Burdon JJ (2003). Leaf scald resistance genes in Hordeum vulgare and Hordeum vulgare ssp spontaneum: parallels between cultivated and wild barley. Aus J Agr Res.

[CR25] Gong P, Riemann M, Dong D (2019). Two grapevine metacaspase genes mediate ETI-like cell death in grapevine defence against infection of Plasmopara viticola. Protoplasma.

[CR26] Grønnerød S, Marøy AG, MacKey J, Tekauz A, Penner GA, Bjørnstad A (2002). Genetic analysis of resistance to barley scald (*Rhynchosporium secalis*) in the Ethiopian line ‘Abyssinian’ (CI668). Euphytica.

[CR27] Hack H, Bleiholder H, Buhr L, Meier U, Schnock-Fricke U, Weber E, Witzenberger A (1992). A uniform code for phenological growth stages of mono- and dicotyledonous plants-extended BBCH scale, general-, Allgemein. Nachrichtenblatt Des Deutschen Pflanzenschutzdienstes.

[CR28] Hanemann A, Schweizer GF, Cossu R (2009). Fine mapping, physical mapping and development of diagnostic markers for the Rrs2 scald resistance gene in barley. Theor Appl Genet.

[CR29] Hofmann K, Silvar C, Casas AM, Herz M, Büttner B, Gracia MP, Contreras-Moreira B, Wallwork H, Igartua E, Schweizer G (2013). Fine mapping of the Rrs1 resistance locus against scald in two large populations derived from Spanish barley landraces. Theor Appl Genet.

[CR30] Huang BE, Verbyla KL, Verbyla AP (2015). MAGIC populations in crops: current status and future prospects. Theor Appl Genet.

[CR31] Huang M, Liu X, Zhou Y, Summers RM, Zhang Z (2019). BLINK: a package for the next level of genome-wide association studies with both individuals and markers in the millions. GigaScience.

[CR32] Huang X, Han B (2014). Natural variations and genome-wide association studies in crop plants. Annu Rev Plant Biol.

[CR33] Kari AG, Griffiths E (1993). Components of partial resistance of barley to *Rhynchosporium secalis*: use of seedling tests to predict field resistance. Ann Appl Biol.

[CR34] Liang P, Liu S, Xu F, Jiang S, Yan J, He Q, Liu W, Lin C, Zheng F, Wang X, Miao W (2018). Powdery mildews are characterized by contracted carbohydrate metabolism and diverse effectors to adapt to obligate biotrophic lifestyle. Front Microbiol.

[CR35] Li HB, Zhou MX (2011). Quantitative trait loci controlling barley powdery mildew and scald resistances in two different barley doubled haploid populations. Mol Breeding.

[CR36] Li MX, Yeung JMY, Cherny SS, Sham PC (2012). Evaluating the effective numbers of independent tests and significant p-value thresholds in commercial genotyping arrays and public imputation reference datasets. Hum Genet.

[CR37] Lipka AE, Tian F, Wang Q (2012). GAPIT: genome association and prediction integrated tool. Bioinformatics.

[CR73] Liu X, Huang M, Fan B, Buckler ES, Zhang Z, Listgarten J (2016). Iterative usage of fixed and random effect models for powerful and efficient genome-wide association studies. PLOS Genet.

[CR38] Looseley ME, Griffe LL, Büttner B, Wright KM, Middlefell-Williams J, Bull H (2018). Resistance to *Rhynchosporium commune* in a collection of European spring barley germplasm. Theor Appl Genet.

[CR39] Looseley ME, Griffe LL, Büttner B (2020). Characterisation of barley landraces from Syria and Jordan for resistance to *Rhynchosporium* and identification of diagnostic markers for Rrs1Rh4. Theor Appl Genet.

[CR40] Looseley ME, Newton AC, Atkins SD (2012). Genetic basis of control of *Rhynchosporium secalis* infection and symptom expression in barley. Euphytica.

[CR41] Mascher M, Gundlach H, Himmelbach A (2017). A chromosome conformation capture ordered sequence of the barley genome. Nature.

[CR42] Mathew B, Léon J, Sannemann W, Sillanpää M (2018). Detection of epistasis for flowering time using bayesian multilocus estimation in a Barley MAGIC population. Genetics.

[CR43] Maurer A, Draba V, Jiang Y (2015). Modelling the genetic architecture of flowering time control in barley through nested association mapping. BMC Genomics.

[CR44] McDonald BA (2015). How can research on pathogen population biology suggest disease management strategies? The example of barley scald (*Rhynchosporium commune*). Plant Pathol.

[CR45] McGrann G, Stavrinides A, Russell J, Corbitt MM, Booth A, Chartrain L, Thomas WTB, Brown JKM (2014). A trade off between mlo resistance to powdery mildew and increased susceptibility of barley to a newly important disease, Ramularia Leaf Spot. J Exp Botany.

[CR46] McLean MS, Hollaway GJ (2018). Suppression of scald and improvements in grain yield and quality of barley in response to fungicides and host-plant resistance. Aus Plant Pathol.

[CR47] Niks R, Parlevliet J, Lindhout P, Bai Y (2011). Breeding crops with resistance to diseases and pests.

[CR48] Novakazi F, Krusell L, Jensen JD, Orabi J, Jahoor A, Bengtsson T, on behalf of the PPP Barley Consortium (2020) You Had Me at “MAGIC”!: four Barley MAGIC populations reveal novel resistance QTL for powdery mildew. Genes 11:1512. 10.3390/genes11121512 10.3390/genes11121512 PMC7766815 33352820

[CR49] Patil V, Bjørnstad Å, Mackey J (2003) Molecular mapping of a new gene Rrs4 CI 11549 for resistance to barley scald (*Rhynchosporium secalis*). Mol Breeding 12:169–183 10.102-3/A:1026076511073

[CR50] Paulitz TC, Steffenson BJ, Ullrich SE (2011). Biotic stress in Barley: disease problems and solutions. Barley: production, improvement and uses.

[CR51] Pickering R, Ruge-Wehling B, Johnston PA, Schweizer G, Ackermann P, Wehling P (2006). The transfer of a gene conferring resistance to scald (*Rhynchosporium secalis*) from Hordeum bulbosum into H. vulgare chromosome 4HS. Plant Breed.

[CR52] Piechota U, Czembor PC, Słowacki P (2019). Identifying a novel powdery mildew resistance gene in a barley landrace from Morocco. J Appl Gen.

[CR53] Rafalski JA (2010) Association genetics in crop improvement. Curr Opin Plant Biol 13:174–180 10.1016/j.pbi.2009.12.004 10.1016/j.pbi.2009.12.004 20089441

[CR54] R Core Team (2017) R: A Language and Environment for Statistical Computing; R Foundation for Statistical Computing: Vienna, Austria

[CR55] Reddy ASN, Ali GS, Celesnik H, Day IS (2011). Coping with stresses: roles of Calcium and Calcium/Calmodulin-regulated gene expression. Plant Cell.

[CR56] Revelle W (2020) psych: procedures for Personality and Psychological Research. In: https://CRAN.R-project.org/package=psych

[CR57] Rohe M, Gierlich A, Hermann H, Hahn M, Schmidt B, Rosahl S (1995). The race-specific elicitor, NIP1, from the barley pathogen, *Rhynchosporium secalis*, determines avirulence on host plants of the Rrs1 resistance genotype. EMBO J.

[CR58] Scott MF, Ladejobi O, Amer S (2020). Multi-parent populations in crops: a toolbox integrating genomics and genetic mapping with breeding. Heredity.

[CR59] Segura V, Vilhjálmsson BJ, Platt A (2012). An efficient multi-locus mixed-model approach for genome-wide association studies in structured populations. Nat Genet.

[CR60] Steiner-Lange S, Fischer A, Boettcher A, Rouhara I, Liedgens H, Schmelzer E (2003). Differential defense reactions in leaf tissues of barley in response to infection by *Rhynchosporium secalis* and to treatment with a fungal avirulence gene product. Mol Plan Microbe.

[CR61] Van Leur JAG, Ceccarelli S, Grando S (1989). Diversity for disease resistance in barley landraces from Syria and Jordan. Plant Breed.

[CR62] Van Raden PM (2008). Efficient methods to compute genomic predictions. J Dairy Sci.

[CR63] vant’t Slot KAE, Gierlich A, Knogge, W, (2007) A single binding site mediates resistance- and disease-associated activities of the effector protein NIP1 from the Barley pathogen *Rhynchosporium secalis*. Plant Physiol 144:1654–1666. 10.1104/pp.106.094912 10.1104/pp.106.094912 PMC1914146 17478637

[CR64] Wang Y, Gupta S, Wallwork H, Zhang XQ, Zhou G, Broughton S (2014). Combination of seedling and adult plant resistance to leaf scald for stable resistance in barley. Mol Breeding.

[CR65] Wallwork H, Grcic M, Li CD, Hayden MJ, Chalmers K, Mather DE (2014) Use of specific differential isolates of *Rhynchosporium commune* to detect minor gene resistance to leaf scald in barley seedlings. Aus Plant Path 43:197–203

[CR66] Williams RJ, Owen H (1975) Susceptibility of barley cultivars to leaf blotch and aggressiveness of *Rhynchosporium secalis* races. T Br Mycol Soc 65:109–114

[CR67] Zaffarano PL, McDonald BA, Linde CC (2011). Two new species of *Rhynchosporium*. Mycologia.

[CR68] Zantinge J, Xue S, Holtz M (2019). The identification of multiple SNP markers for scald resistance in spring barley through restriction-site associated sequencing. Euphytica.

[CR69] Zhang Z, Ersoz E, Lai CQ (2010). Mixed linear model approach adapted for genome-wide association studies. Nat Genet.

[CR70] Zhang YM, Jia Z, Dunwell JM (2019). Editorial: The applications of new multi-locus gwas methodologies in the genetic dissection of complex traits. Front Plant Sci.

[CR71] Zhang X, Ovenden B, Milgate A (2020). Recent insights into barley and *Rhynchosporium commune* interactions. Mol Plant Pathol.

