# Supplementary material for: Pyramiding of scald resistance genes in four spring barley MAGIC populations
Source: Theor Appl Genet. 2021 Aug 4;134(12):3829–43. doi: 10.1007/s00122-021-03930-y (PMC8580920; doi:10.1007/s00122-021-03930-y)
Supplement: Supplementary file 1 — Supplementary material 1 (PDF 175 kb) [file 122_2021_3930_MOESM1_ESM.pdf]

Online Resource 1. Description and correlations in phenotypic data

Article title: Pyramiding of scald resistance genes in four spring barley MAGIC populations.

Journal name: Theoretical and Applied Genetics.

Author names: Juho Hautsalo, Fluturë Novakazi, Marja Jalli, Magnus Göransson, Outi Manninen, Mika Isolahti, Lars Reitan, Stein Bergersen, Lene Krusell, Charlotte Damsgård Robertsen, Jihad Orabi, Jens Due Jensen, Ahmed Jahoor, Therése Bengtsson and the PPP Barley consortium

Affiliation and e-mail address of the corresponding author: [2] Department of Plant Breeding, Swedish University of Agricultural Sciences, P.O. Box 101, 23053 Alnarp, Sweden, therese.bengtsson@slu.se

Contents

Box plots for all observations and populations

Descriptive statistics for all observations and populations

Correlation plots for all observations and populations

Results from statistical analysis for all environments and populations.

Distribution of scald scores in the screenings made to separate MAGIC populations. Scale 1-9 (1= healthy plant, 9=plants are 100% symptomatic/dead). First three letters indicate location (Jok=Jokioinen, Finland, Kor=Korpa, Iceland, Sot=Sotkamo, Finland). First two numbers indicate the year (2017-2019), third number represents the observation time during the season and last number tells the replicate within the trial.

MAGIC 1 + 2

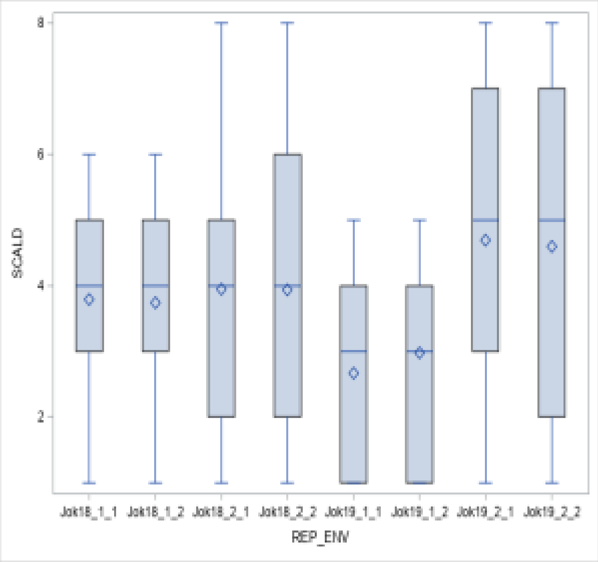

MAGIC 3

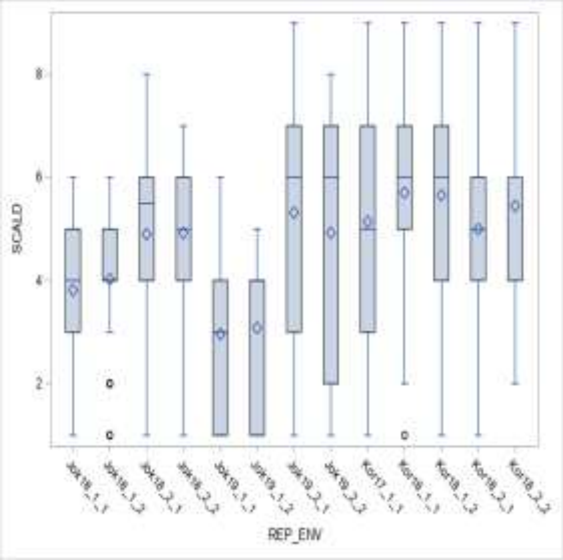

MAGIC 4

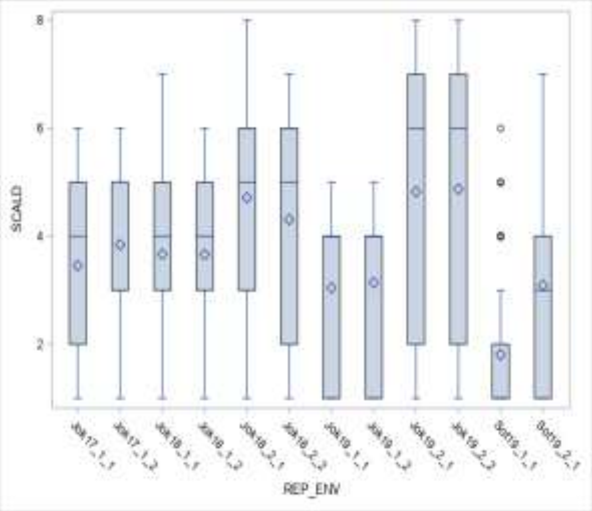

MAGIC 1 to 4

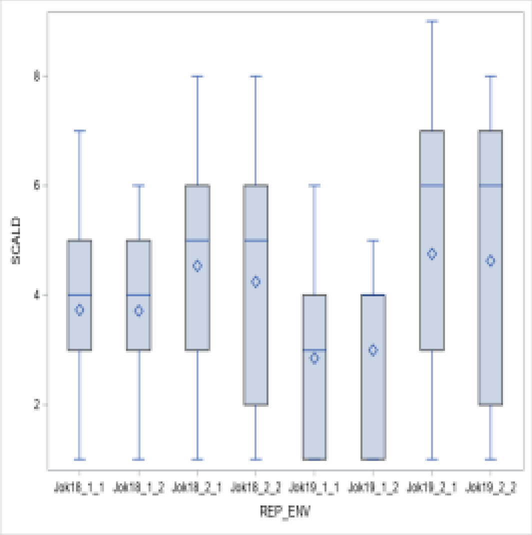

Descriptive statistics for observations by MAGIC populations, computed with psych software package v. 2.0.9 (Revelle, 2020) by R software. Table shows number of observed genotypes (n), mean, standard deviation (sd), median, minimum (min), maximum (max), range, skewness (skew), kurtosis and standard error (se).

MAGIC 1+2

Observation	n	mean	sd	median	min	max	range	skew	kurtosis	se
Jok18_1	134	3.77	1.28	4	1	6	5	-0.8	-0.44	0.11
Jok18_2	134	3.93	1.85	4	1	8	6	-0.11	-1.15	0.16
Jok19_1	134	2.82	1.27	3	1	5	4	-0.12	-1.32	0.11
Jok19_2	134	4.64	2.25	5	1	8	7	-0.19	-1.37	0.19
All	134	3.79	1.5	1.67	1	7	6	-0.31	-1.06	0.13

MAGIC 3

Observation	n	mean	sd	median	min	max	range	skew	kurtosis	se
Jok18_1	82	3.91	1.16	4	1	6	5	-1.04	0.15	0.13
Jok18_2	82	4.94	1.71	5.5	1	8	7	-0.82	-0.34	0.19
Jok19_1	83	3.02	1.46	3.5	1	5	4	-0.22	-1.57	0.16
Jok19_2	83	5.14	2.29	6	1	9	8	-0.36	-1.43	0.25
Kor17_1	83	5.14	2.04	5	1	9	8	-0.07	-0.6	0.22
Kor18_1	83	5.72	1.77	6	1	9	8	-0.4	-0.52	0.19
Kor18_2	83	5.17	1.51	5	2	9	7	0.26	-0.44	0.17
All	83	4.73	1.42	4.93	1	7	6	-0.34	-1.07	0.16

MAGIC 4

Observation	n	mean	sd	median	min	max	range	skew	kurtosis	se
Jok17_1	279	3.65	1.52	4.5	1	6	5	-0.79	-0.9	0.09
Jok18_1	279	3.66	1.25	4	1	7	6	-0.96	-0.23	0.07
Jok18_2	279	4.53	1.91	5	1	8	7	-0.71	-0.87	0.11
Jok19_1	279	3.1	1.33	3.5	1	5	4	-0.5	-1.2	0.08
Jok19_2	279	4.85	2.28	6	1	8	7	-0.72	-1.13	0.14
Sot19_1	279	1.81	1.05	1	1	6	5	1.33	1.27	0.06
Sot19_2	278	3.09	1.69	3	1	7	6	0.21	-1.04	0.1
All	279	3.53	1.4	4.07	1	6	5	-0.6	-0.99	0.08

MAGIC 1 to 4

Observation	n	mean	sd	median	min	max	range	skew	kurtosis	se
Jok18_1	489	3.74	1.24	4	1	7	6	-0.93	-0.2	0.06
Jok18_2	489	4.44	1.89	5	1	8	7	-0.56	-0.98	0.09
Jok19_1	490	3.01	1.34	3.5	1	5	4	-0.33	-1.33	0.06
Jok19_2	490	4.83	2.28	6	1	9	8	-0.51	-1.25	0.1
All	490	4.01	1.54	4.5	1	7	6	-0.62	-0.95	0.07

Spearman's correlations across observations for separate MAGIC populations. Correlations are calculated based on mean values of replicates of a certain line. All are statistically significant at p-value <0.001.

MAGIC 1+2

	Jok18_1	Jok18_2	Jok19_1	Jok19_2
Jok18_1	1			
Jok18_2	0.82	1		
Jok19_1	0.61	0.61	1	
Jok19_2	0.71	0.74	0.87	1

MAGIC 3

	Jok18_1	Jok18_2	Jok19_1	Jok19_2	Kor17_1	Kor18_1	Kor18_2
Jok18_1	1						
Jok18_2	0.79	1					
Jok19_1	0.63	0.60	1				
Jok19_2	0.65	0.66	0.85	1			
Kor17_1	0.53	0.52	0.66	0.64	1		
Kor18_1	0.52	0.60	0.69	0.69	0.69	1	
Kor18_2	0.47	0.47	0.63	0.72	0.63	0.70	1

MAGIC 4

	Jok17_1	Jok18_1	Jok18_2	Jok19_1	Jok19_2	Sot19_1	Sot19_2
Jok17_1	1						
Jok18_1	0.68	1					
Jok18_2	0.72	0.80	1				
Jok19_1	0.74	0.66	0.68	1			
Jok19_2	0.74	0.73	0.78	0.80	1		
Sot19_1	0.60	0.56	0.55	0.59	0.60	1	
Sot19_2	0.73	0.68	0.70	0.73	0.75	0.85	1

MAGIC 1 to 4

	Jok18_1	Jok18_2	Jok19_1	Jok19_2
Jok18_1	1			
Jok18_2	0.79	1		
Jok19_1	0.63	0.66	1	
Jok19_2	0.71	0.75	0.85	1

Summary of statistics for all environments combined (META-R, Alvarado et al. 2020). Environments (year*experiment combinations) were considered random whereas genotypes, replicates, and genotype by environment interactions were considered, as fixed effects.

	MAGIC 1+2	MAGIC 3	MAGIC 4	MAGIC 1 to 4
Broad sense heritability	0.784362	0.805721	0.907517	0.793791
Residual Variance	1.487652	1.494834	1.268867	1.424479
Grand Mean	3.793865	4.65845	3.906369	4.004958
Least Significant Difference	1.634203	1.511467	1.053882	1.64453
Coefficient of Variation	32.14909	26.24551	28.83599	29.80094
number of Replicates	2	2	2	2
number of Environments	2	3	3	2
Genotype significance*	0	0	0	0
GenxEnv significance	1.5E-06	1.77E-11	0.086187	5.3E-81
Env significance	0.74736	0.00571	0.369949	0.317448

*) so close to zero that it is reported as 0.
