# Supplementary material for: Pyramiding of scald resistance genes in four spring barley MAGIC populations
Source: Theor Appl Genet. 2021 Aug 4;134(12):3829–43. doi: 10.1007/s00122-021-03930-y (PMC8580920; doi:10.1007/s00122-021-03930-y)
Supplement: Supplementary file 2 — Supplementary material 2 (PDF 2,519 kb) [file 122_2021_3930_MOESM2_ESM.pdf]

Online Resource 2

Content

QQ-plots and Manhattan plots for MAGIC populations 1+2 (light blue), 3 (golden), 4 (green) and the combined MAGIC 1 to 4 (red) for different models and covariate combinations. SNP density scale (SNPs/ 1Mb) provided in the right corner of each Manhattan plot.

Models tested:
GLM – General Linear Model (Zhang et al. 2010)
MLM – Mixed Linear Model (Zhang et al. 2010)
MLMM – Multiple Loci Mixed linear Model (Segura et al. 2012)
FarmCPU – Fixed and random model Circulating Probability Unification (Liu et al. 2016)
Blink - Bayesian-information and Linkage-disequilibrium Iteratively Nested Keyway (Blink, Huang et al. 2019)
Covariates used:
K – Kinship matrix based on VanRaden Method (Van Raden 2008)
Q – Ancestry coefficient data obtained from STRUCTURE analysis (Pritchard et al. 2000)

Article title: Pyramiding of scald resistance genes in four spring barley MAGIC populations.

Journal name: Theoretical and Applied Genetics.

Author names: Juho Hautsalo, Fluturë Novakazi, Marja Jalli, Magnus Göransson, Outi Manninen, Mika Isolahti, Lars Reitan, Stein Bergersen, Lene Krusell, Charlotte Damsgård Robertsen, Jihad Orabi, Jens Due Jensen, Ahmed Jahoor, Therése Bengtsson and the PPP Barley consortium

Affiliation and e-mail address of the corresponding author: Department of Plant Breeding, Swedish University of Agricultural Sciences, P.O. Box 101, 23053 Alnarp, Sweden, therese.bengtsson@slu.se

MAGIC 1 to 4

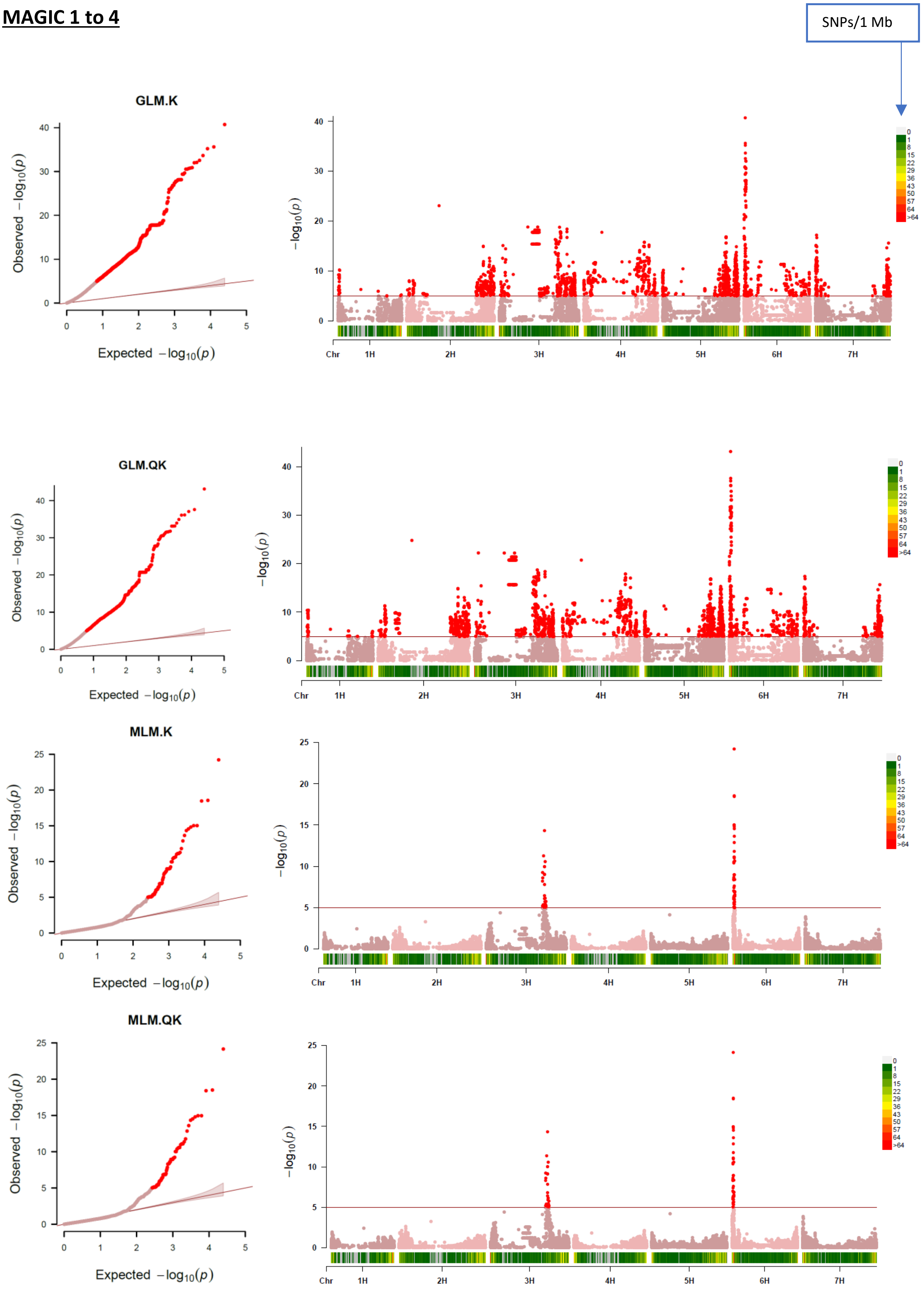
SNPs/1 Mb
GLM.K
GLM.QK
MLM.K
MLM.QK
Observed $-\log_{10}(p)$
Expected $-\log_{10}(p)$
$-\log_{10}(p)$
Chr
1H
2H
3H
4H
5H
6H
7H
0
1
8
15
22
29
36
43
50
57
64
>64

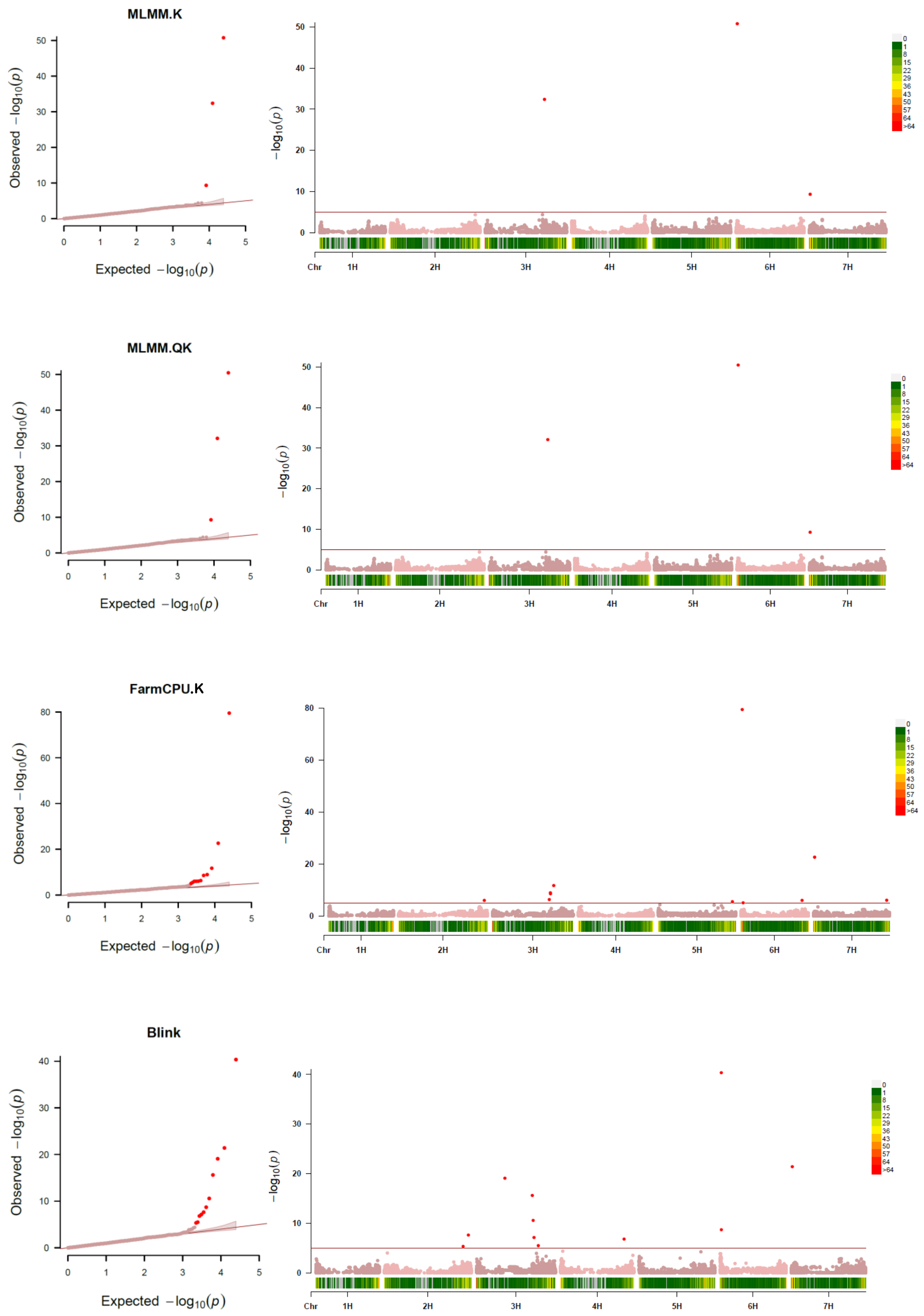

MLMM.K
Observed −log10(p)
Expected −log10(p)
−log10(p)
Chr
1H
2H
3H
4H
5H
6H
7H
0
1
8
15
22
29
36
43
50
57
64
>64
MLMM.QK
FarmCPU.K
Blink

MAGIC 1+2

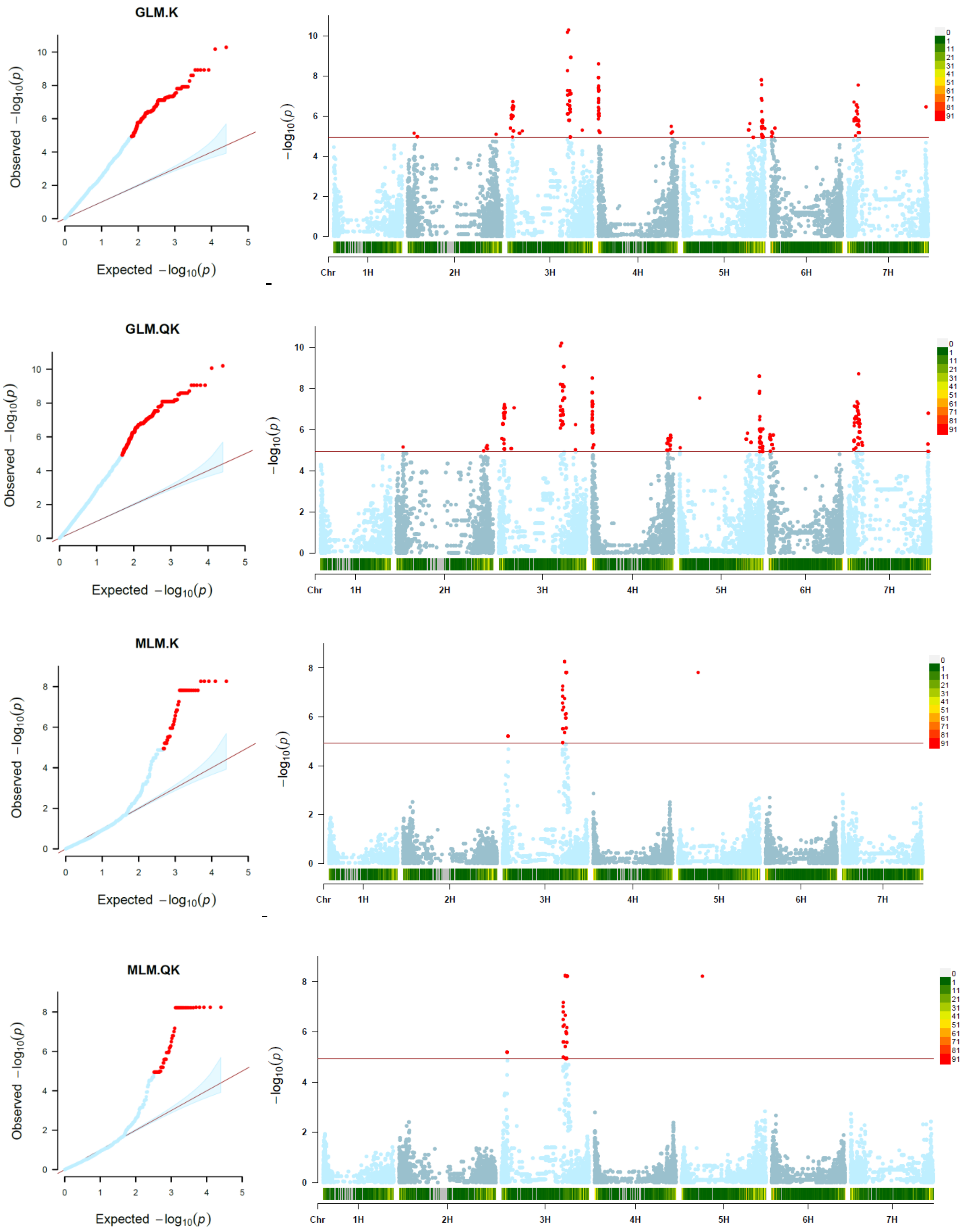
GLM.K
GLM.QK
MLM.K
MLM.QK
Observed $-\log_{10}(p)$
Expected $-\log_{10}(p)$
$-\log_{10}(p)$
Chr
1H
2H
3H
4H
5H
6H
7H

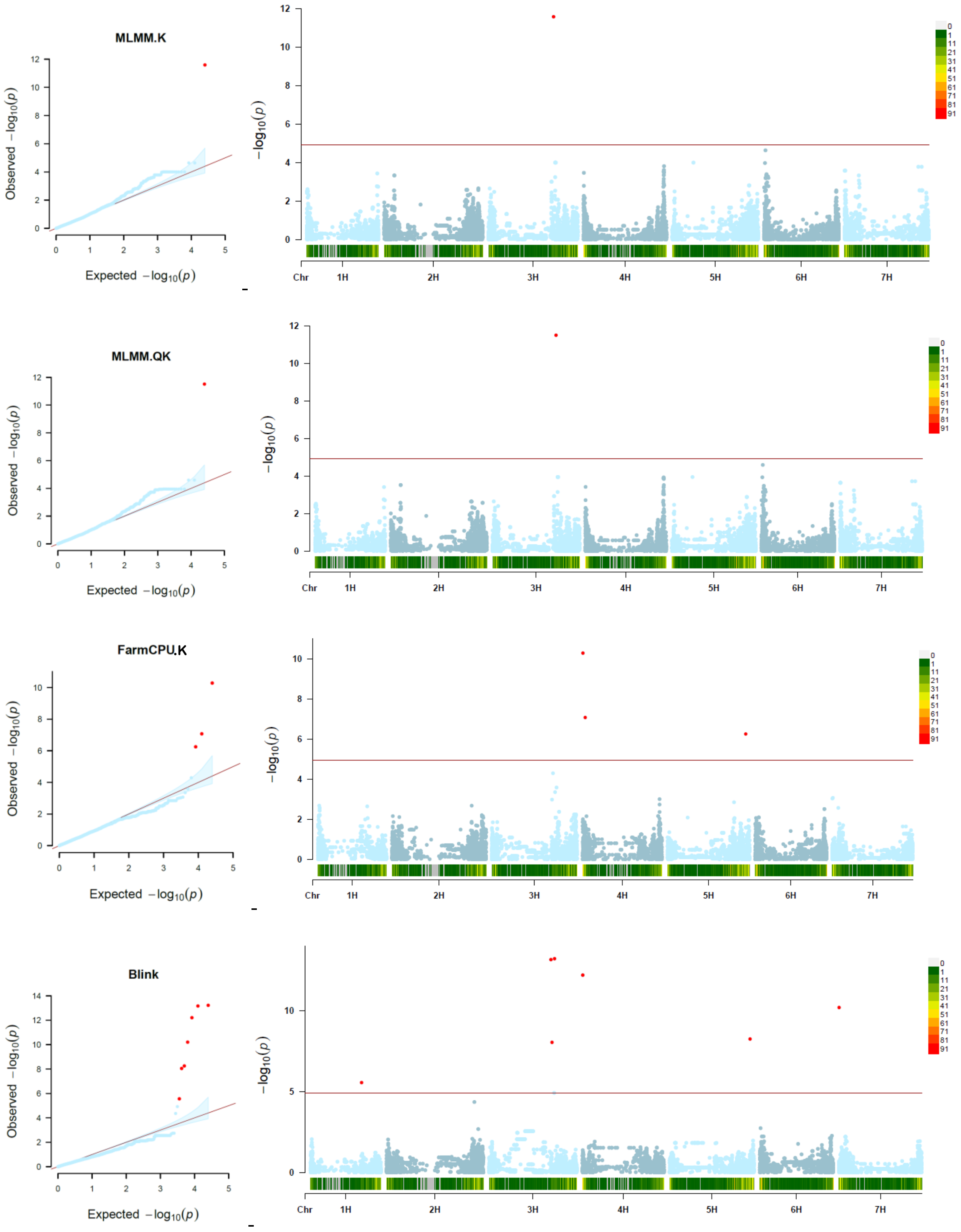
MLMM.K
Observed −log10(p)
Expected −log10(p)
−log10(p)
Chr
1H
2H
3H
4H
5H
6H
7H
0
1
11
21
31
41
51
61
71
81
91
MLMM.QK
FarmCPU.K
Blink

MAGIC 3

GLM.K

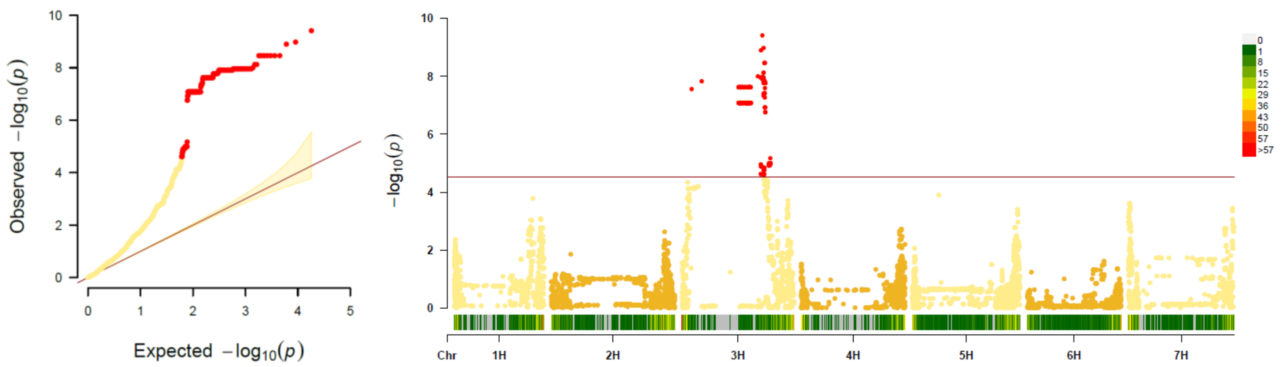

GLM.QK

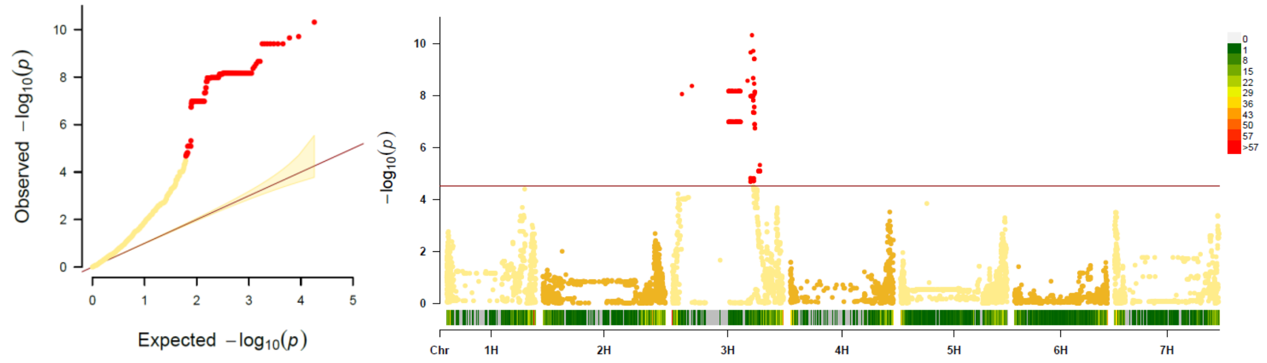

MLM.K

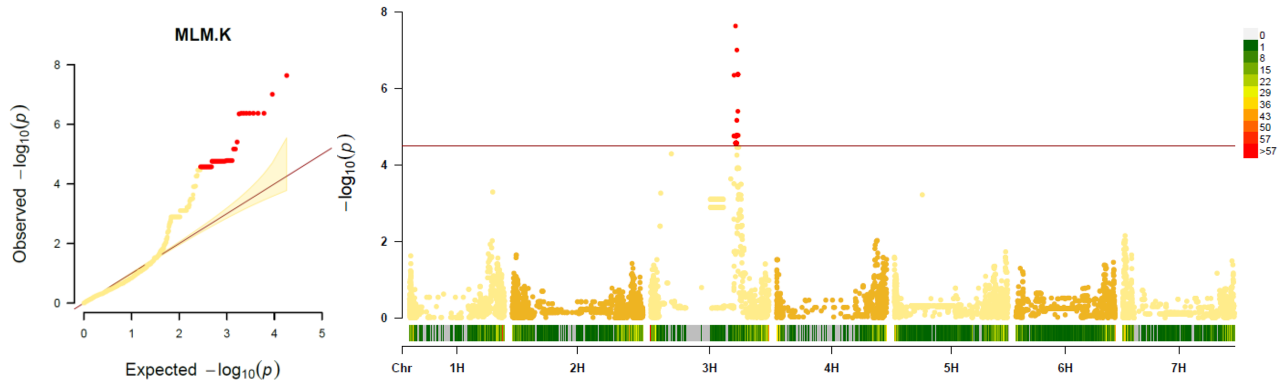

MLM.QK

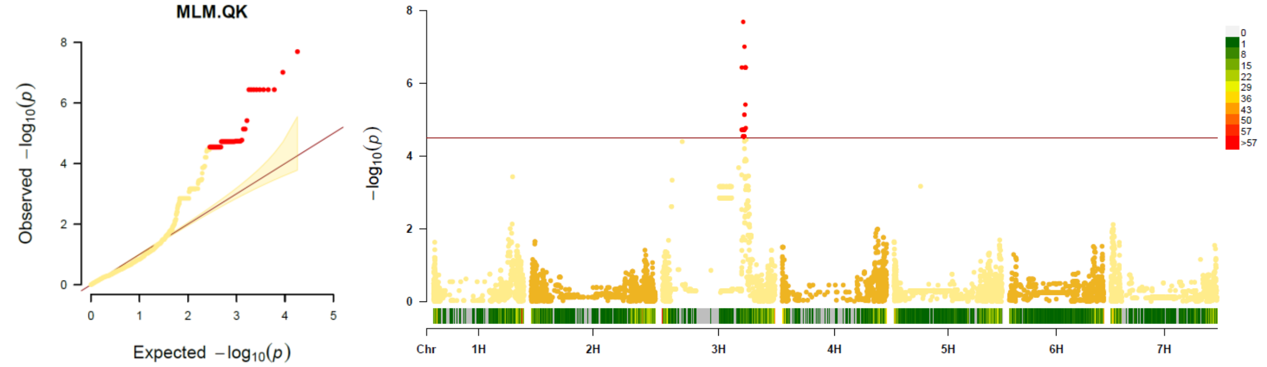

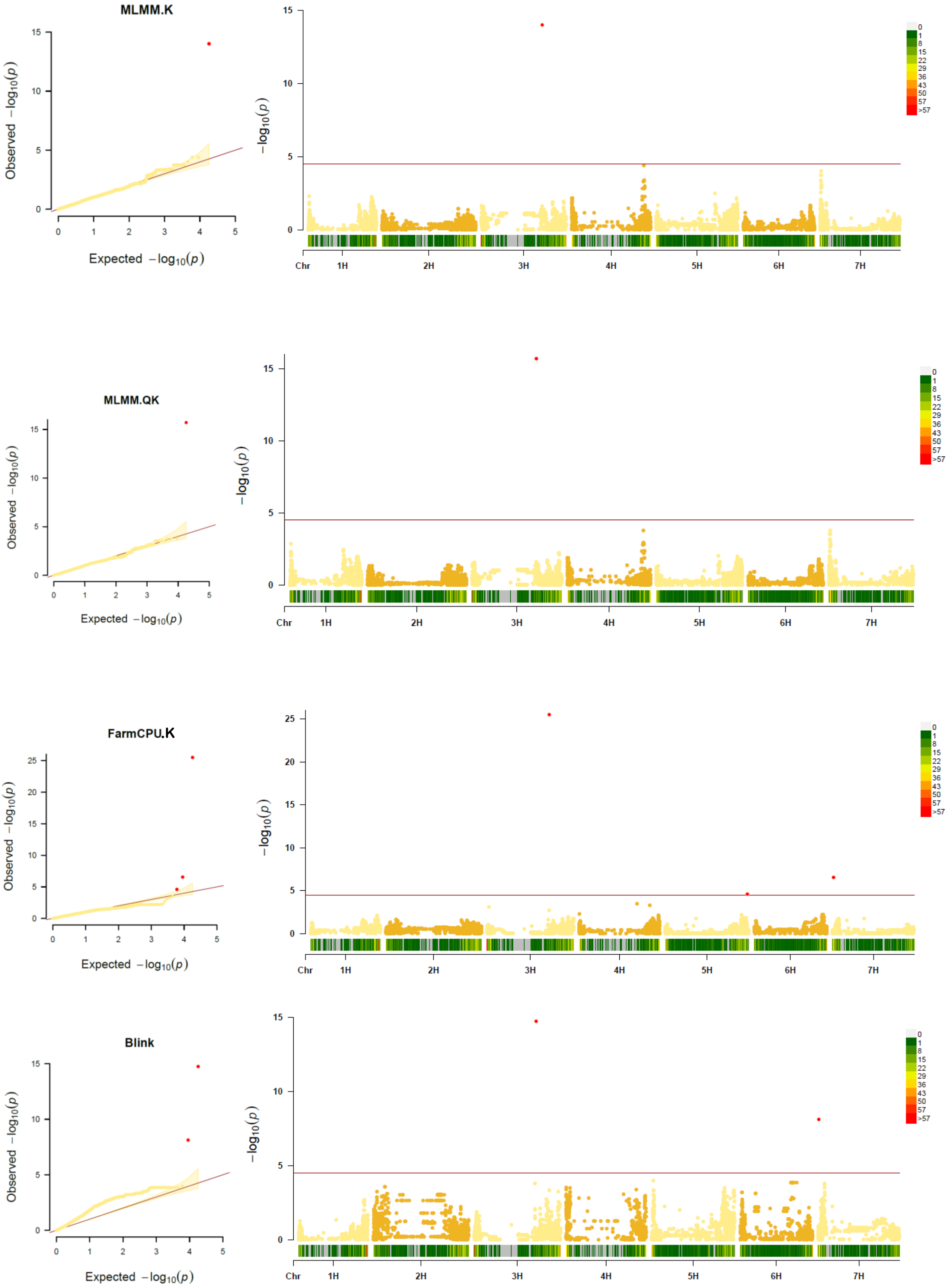

MLMM.K
MLMM.QK
FarmCPU.K
Blink
Observed −log10(p)
Expected −log10(p)
−log10(p)
Chr
1H
2H
3H
4H
5H
6H
7H
0
1
8
15
22
29
36
43
50
57
>57

MAGIC 4

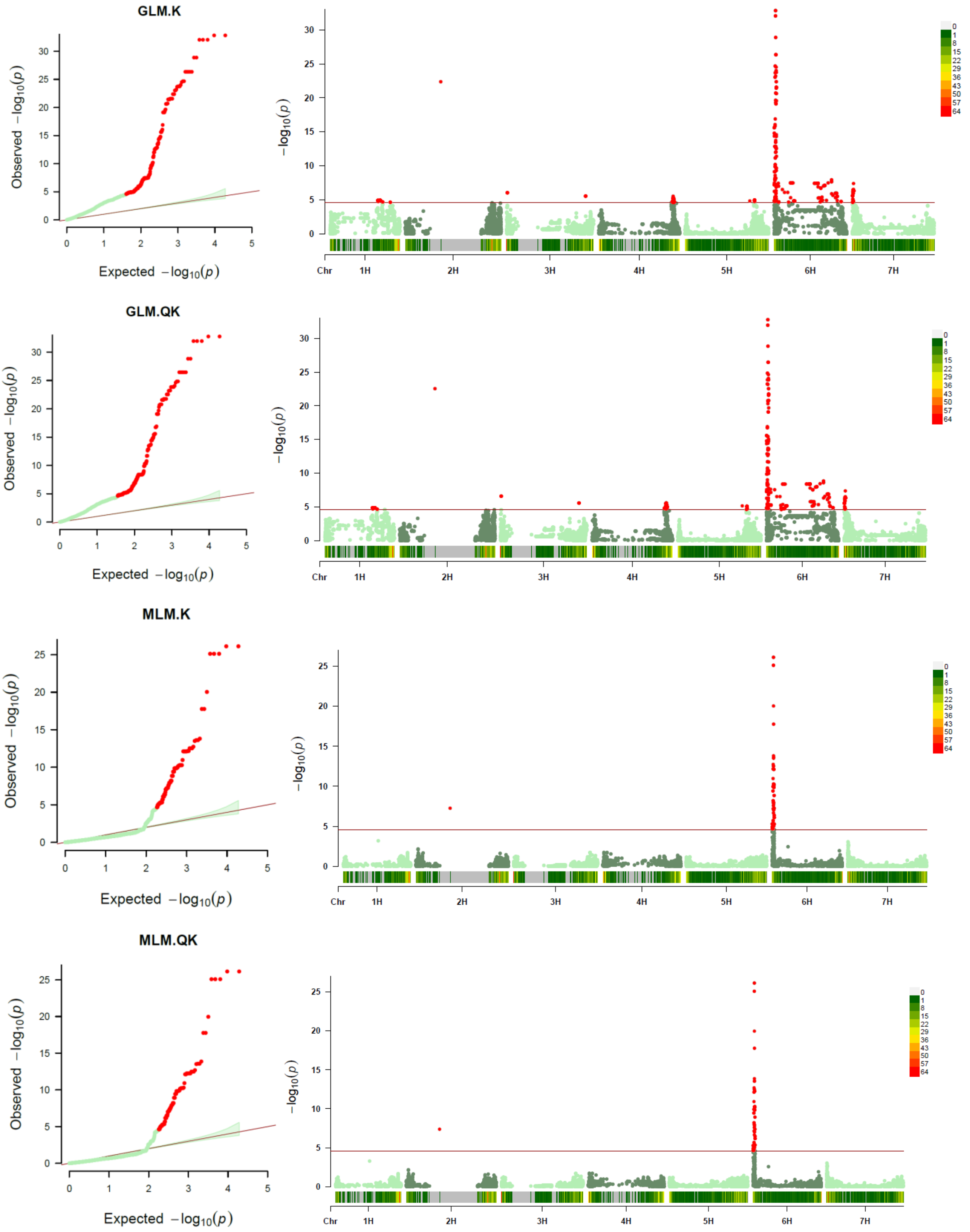
GLM.K
GLM.QK
MLM.K
MLM.QK
Observed −log10(p)
Expected −log10(p)
−log10(p)
Chr
1H
2H
3H
4H
5H
6H
7H
0
1
8
15
22
29
36
43
50
57
64

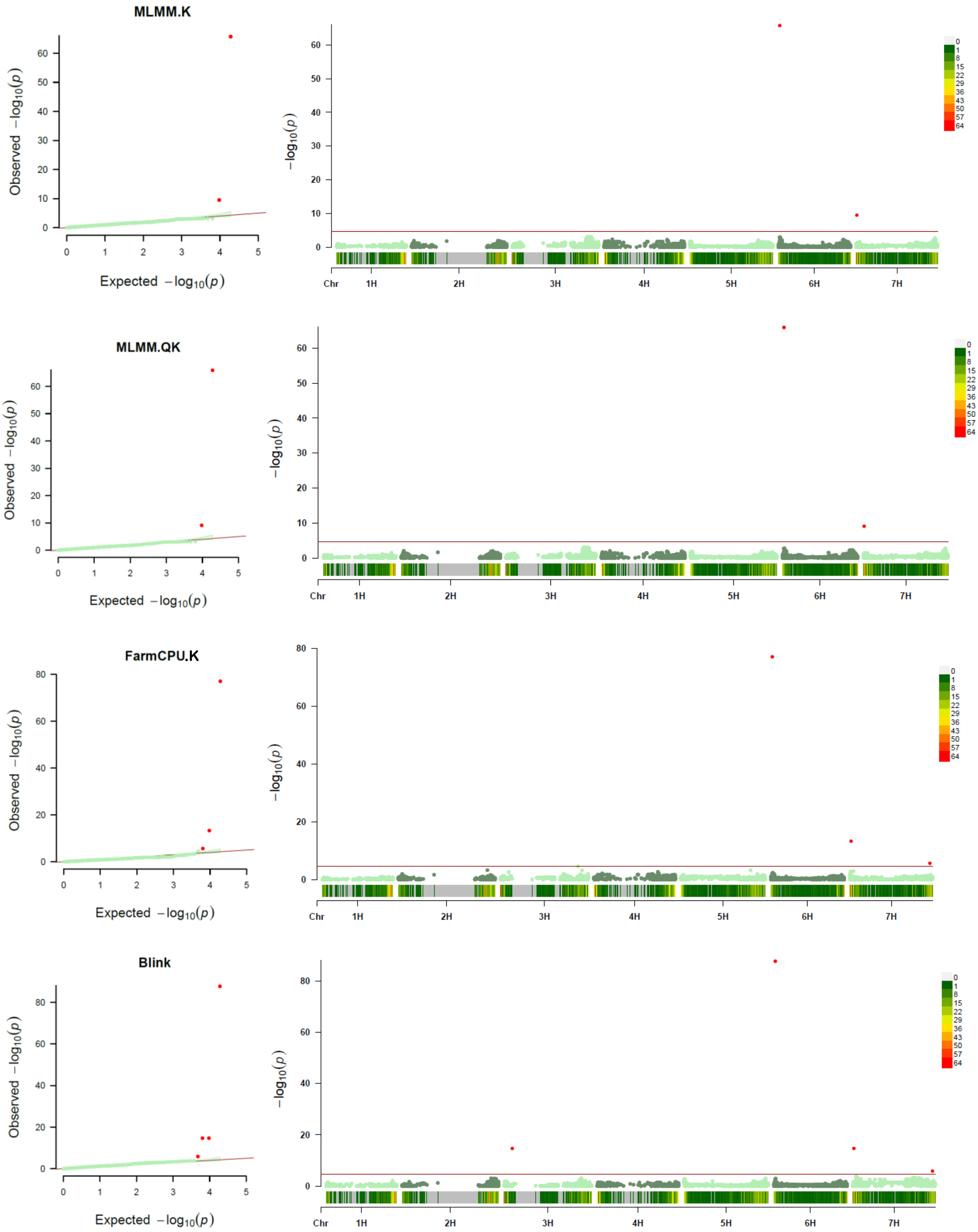
MLMM.K
MLMM.QK
FarmCPU.K
Blink
Observed −log10(p)
Expected −log10(p)
−log10(p)
Chr
1H
2H
3H
4H
5H
6H
7H
0
1
8
15
22
29
36
43
50
57
64
